# Supplementary material for: School-age outcomes among IVF-conceived children: A population-wide cohort study
Source: PLoS Med. 2023 Jan 24;20(1):e1004148. doi: 10.1371/journal.pmed.1004148 (PMC9873192; doi:10.1371/journal.pmed.1004148)
Supplement: S1 Fig — (DOCX) [file pmed.1004148.s001.docx]

**Fig 1 – Direct Acyclic Graph (DAG)**


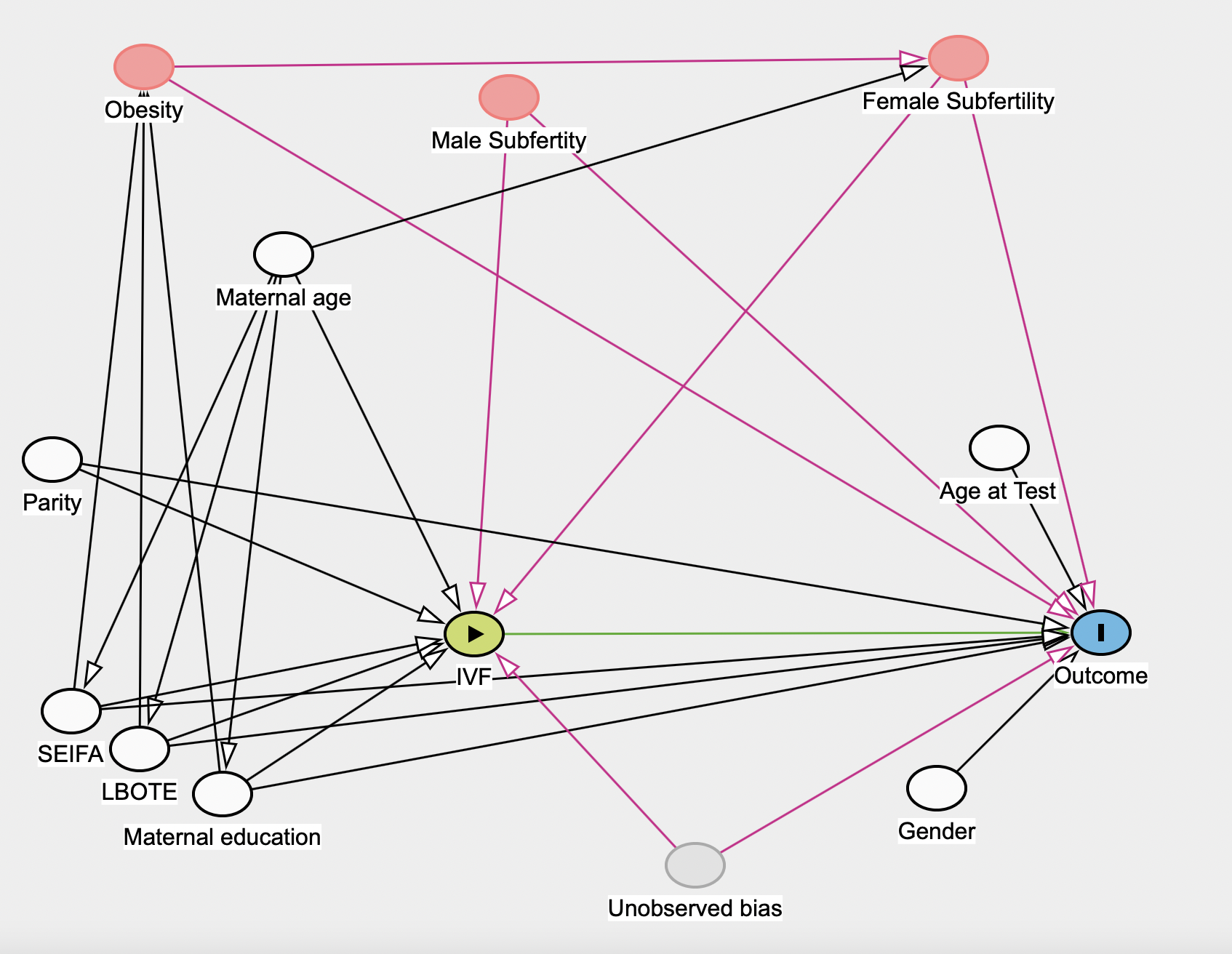


Abbreviations: SEIFA – Socioeconomic index for Areas, LBOTE – Language background other than English,

IVF – Invitro Fertilisation

Exposure

Covariates (included in model

Covariates (not included in model)

Outcome
