# Supplementary material for: School-age outcomes among IVF-conceived children: A population-wide cohort study
Source: PLoS Med. 2023 Jan 24;20(1):e1004148. doi: 10.1371/journal.pmed.1004148 (PMC9873192; doi:10.1371/journal.pmed.1004148)
Supplement: S1 File — (DOCX) [file pmed.1004148.s002.docx]

**Description of Outcome Metrics**

**Australian Early Developmental Census (AEDC)**

The AEDC is a validated research tool performed every three years across all schools in Australia.^1^ The assessment on the students aged 4-6 years is performed by school teachers, who complete a 96 item standardised questionnaire, based on their knowledge of the child during the first few months of school. The tool is structured to assess a child’s broad functional development across five domains: physical health and wellbeing, social competence, emotional maturity, language and cognitive skills (school-based) and communication skills and general knowledge. Each domain is scored out of 10 and the results categorised into ‘developmental vulnerability’ (score less than 10^th^ centile), ‘at risk’ (score 10-25^th^ centile) or ‘on track’ (score > 25^th^ centile); the AEDC domain scores equating to each category were established during the 2009 data collection. In the case of a child who has been identified as having special needs, a AEDC domain score is calculated but no domain category is allocated. Global measures of developmental vulnerability are also reported. Each domain and the global measures have been established to correlate with later school performance.^1-3^

**The National Assessment Program – Literacy and Numeracy (NAPLAN)**

NAPLAN is an annual school-based psychometric assessment, performed nationwide by students in grades 3, 5, 7 and 9 across five educational domains: grammar and punctuation, reading, writing, spelling and numeracy.^4^ Student progress is assessed relative to their current year level. Our study focused on children in their 4^th^ year of primary school (Grade 3), aged 7-9 years. Students receive an individual scaled score for each domain. This scale score can be converted to a standardised z-score using the published national mean scores for each year of the test to account for year-to-year variation in test performance. An overall z-score was calculated and used as the primary outcome, with the individual domain z-scores examined as secondary outcomes.

**References**

1. Brinkman SA, Silburn S, Lawrence D, Goldfeld S, Sayers M, Oberklaid F. Investigating the Validity of the Australian Early Development Index. *Early Education and Development*. 2007/10/11 2007;18(3):427-451. doi:10.1080/10409280701610812

2. Brinkman S, Gregory T, Harris J, Hart B, Blackmore S, Janus M. Associations Between the Early Development Instrument at Age 5, and Reading and Numeracy Skills at Ages 8, 10 and 12: a Prospective Linked Data Study. *Child Indicators Research*. 2013/12/01 2013;6(4):695-708. doi:10.1007/s12187-013-9189-3

3. Brinkman SA, Gregory TA, Goldfeld S, Lynch JW, Hardy M. Data resource profile: the Australian early development index (AEDI). *Int J Epidemiol*. Aug 2014;43(4):1089-96. doi:10.1093/ije/dyu085

4. ACARA - Australian Circulum and Reporting Authority. Test development. <https://www.nap.edu.au/about/test-development>
