# Supplementary material for: School-age outcomes among IVF-conceived children: A population-wide cohort study
Source: PLoS Med. 2023 Jan 24;20(1):e1004148. doi: 10.1371/journal.pmed.1004148 (PMC9873192; doi:10.1371/journal.pmed.1004148)
Supplement: S2 File — (DOCX) [file pmed.1004148.s003.docx]

**– Life After the Lab –**

**What is the causal effect of mode of conception on primary school-aged developmental and educational outcomes for children?**

**PRE-SPECIFIED STATISTICAL ANALYSIS PLAN**

**Version 11.0, May 2020**

**RESEARCH TEAM – roles, responsibilities and signatures**

**Dr Amber Kennedy ____________approved 04/05/2020**

PhD Scholar, FRANZCOG (Mercy Perinatal/University of Melbourne)

**Dr Anthea Lindquist ____________approved 05/05/2020**

Perinatal Epidemiologist, FRANZCOG (Mercy Perinatal/University of Melbourne)

**Professor Stephen Tong ____________approved 05/05/2020**

Clinician scientist, FRANZCOG (Mercy Perinatal/University of Melbourne)

**Professor Susan Walker ____________approved 04/05/2020**

Maternal fetal medicine specialist, FRANZCOG (Mercy Perinatal/University of Melbourne)

**Professor Beverley Vollenhoven ____________approved 04/05/2020**

Reproductive endocrinologist/IVF specialist, FRANZCOG (Monash IVF)

**Associate Professor Kate Stern ____________approved 04/05/2020**

Reproductive endocrinologist/IVF specialist, FRANZCOG (Melbourne IVF)

**Professor Jeanie Cheong ____________approved 05/05/2020**

Academic neonatologist, FRACP (Royal Women’s Hospital, University of Melbourne)

**Dr Roxanne Hastie**

Perinatal Epidemiologist, PhD (Mercy Perinatal/University of Melbourne)

**Dr Jon Quach ____________approved 04/05/2020**

Educationalist academic, PhD (Department of Education, University of Melbourne)

**Dr Richard Hiscock ____________approved 04/05/2020**

Statistician and anaesthetist, ANZCA (Mercy Hospital for Women)

**Professor Lyle Gurrin**

Biostatistician and Project Statistical Advisor, PhD (university of Melbourne)

**BACKGROUND**

**In-Vitro Fertilisation (IVF) is common.** 5% of Australian births are now conceived by IVF. It is well established that pregnancy following IVF is associated with higher risk of maternal and perinatal complications than pregnancy where conception occurs naturally. There is emerging epidemiological evidence suggesting that Intra-Cytoplasmic Sperm Injection (ICSI) may be associated with an increased risk of congenital abnormalities, Autism Spectrum Disorder and other developmental abnormalities compared to (standard) IVF or natural conception. Analysis of existing data has, however, generated results on the risk of these adverse outcomes that are not consistent across studies.

**There are currently few published studies looking at population-wide school-age education and developmental outcomes among children following IVF conception with appropriate comparison between difficerent types of assisted reproductive technology (ART) and a control group representing natural conception (NC).** We will use record-linkage of existing administrative datasets to investigate the evidence of a causal relationship between relatively rare exposure of IVF (compared to natural conception, or ICSI compared to IVF) and the developmental and educational outcomes of primary school-aged children. Outcomes measures of primary interest are achievement assessed using the Australian Early Developmental Census (AEDC) and National Assessment, Literacy and Numeracy (NAPLAN) metrics.

**CAUSAL RESEARCH QUESTION**

***What are the causal effects of Invitro Ferilization (IVF) v Natural Conception (NC) and IVF with Intracytoplasmic Sperm Injection (ICSI) v conventional IVF on the school-age developmental and educational outcomes for children?***

P – All Mothers (and their babies) who gave birth in Victoria during the period 2005-2018

*Note: we intend to consider mothers, fathers and children part of the target population even if we know that they do not have outcomes data available (e.g. AEDC)*

I – Conception via (i) any IVF; (ii) IVF with ICSI.

C – Conception via (i) natural conception (excluding ovulation induction (OI) and intra-uterine insemination (IUI)); (ii) conventional IVF.

O – Developmental and educational outcomes – AEDC and NAPLAN (Grade 3) scores.

T – The nominal age at which children are measured for AEDC (foundation school year: 4-6 years of age) and NAPLAN (school year level 3: 7-9years of age) are typically measured.

**NULL HYPOTHESIS**

**The population distribution of school-aged developmental and educational outcomes for children conceived via conventional IVF or ICSI are the same as those of children born after natural conception.**

**DATA SOURCES**

1. Victorian Perinatal Data Collection
   1. Approximately 1,000,000 Victorian births between 2005-2018
   2. Routinely-collected data from the electronic Birthing Outcome System (BOS).
2. Major IVF providers in Victoria
   1. Cycle data from all pregnancies that resulted in a birth from each of the following IVF providers 2005-2018 (approximately **30,000 cycles):**
      1. Melbourne IVF
      2. Monash IVF
      3. City Fertility Centre
3. Australian Early Developmental Census
   1. Performed *every three years* across Australia. These data are collected by prep school teachers who conduct an observational assessment of every child in their first year of full-time primary school.
   2. Birth data to be linked with corresponding AEDC data from 2009 (unlikely to link with births in our study cohort), **2012, 2015, 2018.**
   3. **Five domains of assessment:**
      1. Physical health and wellbeing – looks at absence from school due to illness, independence with toileting, coordination, fine motor skills and physical ability to climb stairs, and other aspects.
      2. Social competence – assesses ability to get along with peers, respect for adults and others, curiosity about the world and eagerness to play and learn, willingness to help other children who are hurt or having difficulty with an activity and problem-solving day-to-day tasks.
      3. Emotional maturity – aims to identify impulsive or bullying behaviour, as well as the child’s ability to make decisions, be patient and attentive, share and wait their turn in games.
      4. Language and cognitive skills – assesses ability to use English effectively; listen and follow instructions in addition to more in-depth assessments of reading, writing and numeracy.
      5. Communication skills and general knowledge – looks at the child’s ability to tell a story, participate in imaginative play, communicate their own needs to their peers and to adults and assesses their knowledge about the world (eg. the seasons, types of fruit, different animals etc).
   4. From the teacher’s assessment, an individual score is calculated for each child. Outcome measures include *both* a **score** (out of 10) and a **centile** for each domain for every child. Centile scores are used to define:
      1. *developmentally “vulnerable” = <10^th^ centile (in any domain)*
      2. *developmentally “at risk” = 10-25^th^ centile (in any domain)*
      3. *developmentally “on track” = > 25^th^ centile (in any domain)*
4. National Program for Numeracy and Literacy Assessment (NAPLAN) – Victorian Data
   1. Annual school-based assessment
   2. Performed in school years 3, 5, 7, 9
   3. Four domains of assessment, each with a scaled **score** out of 1000, a “band” **category** (1-10 & 1-6 for grade 3) and a dichotomous **“**above or below” **minimum standard**.
      - *Reading*
      - *Writing*
      - *Language conventions (spelling, grammar and punctuation)*
      - *Numeracy*
      - *Overall score – overall academic ability (if data available)*

**DESCRIPTIVE ANALYSIS**

**1. Flow chart of study participants:**


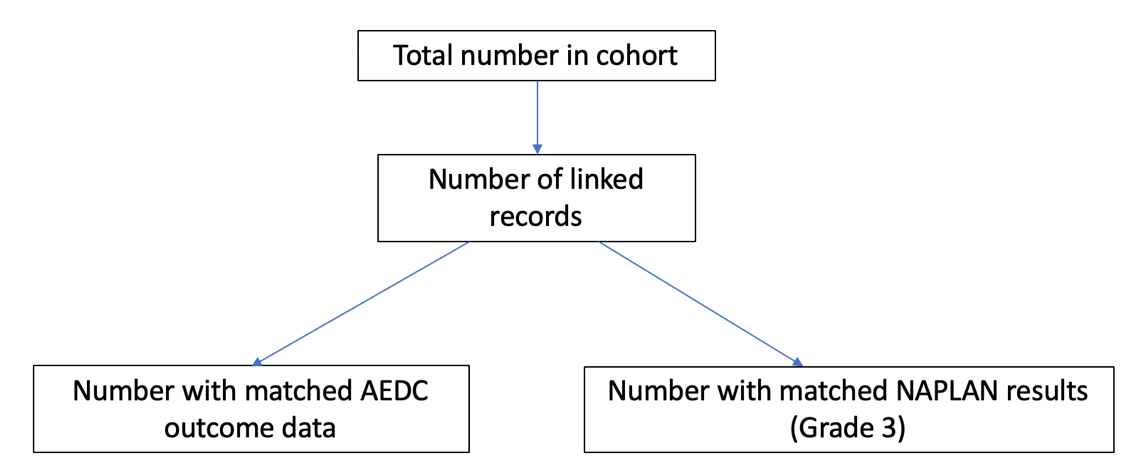


**2. Population characteristics to be described:**

a) Overall cohort

b) Non-IVF cohort (naturally-conceived children) only

c) ART cohort overall (IVF, ICSI, IUI, OI)

d) IVF cohort only

e) ICSI cohort only

| **VARIABLE** | **DESCRIPTOR** |
| --- | --- |
| **Child baseline data** | |
| Sex | (% female) |
| Language Background Other Than English | (%) |
| Aboriginal / Torres Strait Islander | (%) |
| [*Mother born overseas*]  [*Father born overseas*]  Any parent born overseas | (%) |
| Birthweight | Mean and SD |
| Gestational age of delivery | Median and IQR |
| Plurality:  No. Twins  No. Triplets  No. Quads | Absolute numbers and % |
| Method of delivery | (% C/S) |
| **Maternal baseline data** |  |
| Maternal age | Median and IQR |
| Marital status:  Married/de facto  Single  Divorced  Widowed | (% of each category) |
| Level of maternal education:  Year 10 or below  High school  Trade/TAFE/diploma/certificate  Undergraduate Uni degree or above | (% of each category) |
| Maternal Occupation:  Senior management,  Other business manager, Tradesman/woman, clerks, sales and service staff,  Machine operators,  Not in paid work,  Not Stated/Unknown | (% of each category) |
| SEIFA (Socio-Economic Index for Areas) quintile:  1 (most disadvantaged)  2  3  4  5 (least disadvantaged) | (%) for each category |
| **Second parent baseline data** |  |
| Level of second parent education:  Level of maternal education  Year 10 or below  High school  Trade/TAFE/diploma/certificate  Undergraduate Uni degree or above | (% of each category) |
| Second Parent Occupation:  Senior management,  Other business manager, -Tradesman/woman, clerks, sales and service staff,  Machine operators,  Not in paid work,  Not Stated/Unknown | (% of each category) |
| **Childhood outcome data** |  |
| **AEDC** | |
| Individual score | Median (IQR) |
| Developmental vulnerability (<10^th^ in ≥2 domains) | % |
| **NAPLAN** |  |
| Scaled score | Median (IQR) |
| Z-score | Mean (SD) |
| Band category | N (%) |
| Binary – minimum standard | % |
| Public or private school | % (if data available) |

Numbers and characteristics of children for which NAPLAN outcome data was *not* matched or available will also be described given that NAPLAN is not compulsory.

**3. Figures**

a) AEDC:

- Graphs will be generated to describe a) the proportion of developmentally vulnerable children (<10^th^ centile ≥2 domains) and b) the proportion of children ‘on track’ amongst the overall cohort, natural conception, overall ART (assisted reproduction), IVF-only and ICSI-only cohorts

b) NAPLAN:

- Graphs will be generated to describe a) the proportion of children in the lowest band category and b) the proportion of children classified as ‘below minimum standard’ amongst the overall cohort, natural conception, overall ART (assisted reproduction), IVF-only and ICSI-only cohorts
- Represent as box and whisker plot of test scores for each study population
- Use of z-scores standardised by year of test and year level – we will use whole of Australia national standards

**STATISTICAL ANALYSIS**

**PART ONE – AEDC**

**Causal research question:** What is the causal effect of conception via IVF compared to natural conception on AEDC-defined outcomes for children?

**Exposure**: IVF conception.

**Primary outcome:** A score of <10^th^ centile in “2 or more” domains (Binary yes/no).

**Secondary outcomes:** A score in the lowest decile in each domain: 1) physical health and wellbeing, 2) social competence, 3) emotional maturity, 4) language and cognitive skills, and 5) communication skills and general knowledge.

**Inclusion criteria:** Children born in the study time period.

**Exclusion criteria:** Multiple pregnancies, known congential anomaly.

**Causal Inference methodology**

| *The steps to* ***causal inference****:*   1. *Think carefully about the precise nature of the causal question to be addressed, usually around a currently humanly feasible intervention that we wish to apply to the whole of the target population, and compare the outcome (averaged over the whole of the target population) to the corresponding average outcome that occurs when no one in the target population gets the intervention;* 2. *Convert the causal question in Step 1 to a precise quantity to be estimated (a causal estimand), usually using the notation of the potential outcomes approach to causal inference.* 3. *At least state and, if possible, be rigorous and honest about the the assumptions (e.g. consistency, conditional exchangeability, positivity) under which the estimand in Step 2 can be identified from the data at hand;* 4. *Find (or develop!) estimation strategies (i.e. statistical methods) that are valid under the assumptions in Step 3. Apply these to the data in Step 3;* 5. *Use statistical tools to assess quantitatively the sensitivity of the results to plausible departures from the assumptions in Step 3.* |
| --- |

1. Develop a causal model using directed acyclic graphs (DAGs) to both identify and summarize causal pathways.
   1. Potential confounders – demographic and perinatal – will be determined by a quorum of specialists with expert knowledge:
      1. Childs age at assessment (continuous)
      2. Gender (binary)
      3. Birthweight centile (continuous or binary, eg < 10^th^ centile)
      4. Gestational age of delivery (continuous)
      5. Mode of delivery (categorical)
      6. Level of maternal education (categorical)
      7. Maternal age (continuous)
      8. SEIFA quintile (categorical)
      9. LBOTE (binary)
      10. ATSI (binary)
      11. BMI
      12. Other potential confounders:
          1. Parents overseas born
          2. Occupation of both parents
          3. Second parent level of education
          4. Parity

The list of covariates to be included in the propensity score model will be finalised after consideration of potential open backdoor causal paths and conditioning effects of collider nodes. The list above will be used as start. Distinction will be made between the standard epidemiological definition of a confounder and the causal pathway definition.

**Causal pathway and confounding** *(excerpt from Causal Inference , What if. M Hernan & J Robins p85:* [*https://www.hsph.harvard.edu/miguel-hernan/causal-*](https://www.hsph.harvard.edu/miguel-hernan/causal-) *inference-book/*)

“..the bias has the same structure: it is due to the presence of a cause (known covariate L or unknown covariare U) that is shared by the treatment A and the outcome Y, which results in an open backdoor path between A and Y. We refer to the bias caused by shared causes of treatment and outcome as confounding,…”

**These potential confounders (PS_covariates) will then be used in the construction of a propensity score model for exposure assignment (vide infra).**

1. Handling of missing data using Multiple Imputation

a. Consideration of missing data will be confined to all the covariates identified for the construction of a (propensity score) model of the exposure status. When imputing these missing covariate values the imputation model will include include: the outcome; all covariates identified for inclusion in the propensity score model with any transformations and interaction terms where appropriate; as well as additional auxillary covariates* from the BOS database/NAPLAN/AEDC databases. *These co-variates will be selected once the data are available for review.

b. Multiple imputation will be performed using either Multiple Imputation by Chained Equations (MICE) and/or Missing Covariate Indicator Method (MCIM) and the imputed datsets will then be used for propensity score construction. Subjects with missingness greater than 50% of PS_covariates will be excluded from analysis, this proportion will be assessed and if substantial, may result in changes to the PS_covariate list used for ongoing analysis.

c Diagnostics will then be performed to assess the quality of the imputed datasets.

d. For each of these imputed datasets Inverse Probability Weights (IPW, vide infra) will be calculated and used in weighted regression analyses. For each outcome metric, average point estimates and standard errors (95% CI) appropriately adjusted, given the imputation framework, will be obtained. The number of imputed datasets will be set at a minimum of 20 with the number increased to equal to the highest percentage of missingness in the raw PS_covariate list (ceiling of 50% by design). Details of imputation and assessment of adequacy will be formally presented.

1. Development of Inverse Probability Weights.

a) For each imputed dataset, logistic regression with robust standard errors will be performed for the binary exposure against all the PS_covariates. Inverse probability weights will be calculated for each individual given their exposure and these will be used to weight the adjusted regression model.

b) For each imputed dataset the distribution of IPW by exposure status will be examined to ensure: (i) that there is common support (overlap) of the distributions adequacy of adjustment; and (ii) that the weighting was effective in equalizing the covariate distributions in the ‘pseudopopulation’. This will be performed using standardized difference (StD) constructed from the imputed mean minus the raw data mean for all PS_covariate, with an StD of less than 10% being considered adequate. Forrest plots comparing unadjusted and adjusted covariate distributions will also be performed.

1. Regression modelling
   1. All causal regression modelling wil be performed within the multiple imputation frame-work using the IPW propensity score-based weights.
   2. The outcome metric is considered to be absolute risk difference between IVF exposure groups. This will be performed using either generalized linear modelling with Poisson link to directly generate Relative Risks or via logistic regression modelling.
   3. Software: analysis will be performed using Stata v16 (StataCorp. 2019. *Stata Statistical Software: Release 16*. College Station, TX: StataCorp LLC. Causal inference will be performed using teffects commands (Treatment-effects estimation for observational data - see TE -teffects) run within within Stata’s multiple imputation suite.

*The* ***primary analysis*** *will focus on the* ***total effect*** *of IVF conception adjusted for common cause confounders. Part 4 outlines the proposed mediation analysis where* ***direct*** *and* ***indirect*** *(mediated) pathways (eg via prematurity) may be further investigated.*

**PART TWO – NAPLAN**

**Causal research question:** What are the NAPLAN-defined outcomes for children conceived via IVF vs those conceived naturally?

**Exposure:** IVF conception.

**Primary outcome:** Z score to be generated from our cohort using published mean and SD for national-scaled scores.

- Means and SD for each child will be scaled to the national score for the particular year that the child was assessed. The scaled score provided is overall, thus it is expected that children in Grade 3 will have lower average scores than those in Grade 5.

**Secondary outcomes:** Above or below minimum standard for each of the four domains (binary).

**Inclusion criteria:** Children born during the study time period.

**Exclusion criteria:** Multiple pregnancies, known congential anomaly.

**Causal Inference methodology**

The analysis steps 1, 2 and 3 will be identical for that detailed above for Part One (AEDC outcome) and are not repeated here.

**Regression modelling**

- NAPLAN outcome is a continuous z-score.
- Given the cohort size it is very likely that differences in outcome between exposure groups will be highly statistically significant. The *magnitude of outcome difference* that constitutes a clinically meaningful difference is approximated by an effect size of SD 0.1, 0.2 and 0.4 – considered as mild, moderately and extremely clinically significant, respectively.
- Regression will be performed using inverse probability weighted linear regression modelling with continuous z-score as the outcome and IVF as the exposure.
- Post-analysis outcome difference will be translated and presented in a variety of effect size metrics:
  - Grattan institute - year level equivalent (EYL) of scaled score
    - Worked example: The national mean for **reading** in **2019** was 432 ELY = 3, SD was 86 so an effect size of SD of 0.1 = 423, 0.2 = 414.8 and 0.4 = 397.6 a EYL score of 2.81, 2.65 and 2.30 translating to **2**, **4** and **8** months of schooling.
      - Goss, P., and Chisholm, C., 2016, Widening gaps: what NAPLAN tells us about student progress. Technical Report, Grattan Institute

*Again, the* ***primary analysis*** *will focus on the* ***total effect*** *of IVF conception adjusted for common cause confounders. Part 4 outlines the proposed mediation analysis where* ***direct*** *and* ***indirect*** *(mediated) pathways (eg via prematurity) may be further investigated.*

**PART THREE – ICSI ANALYSIS**

ICSI forms an important and growing proportion of IVF technique. Outcomes amongst this sub-population will be explored separately and subsequent to the analyses in Part One and Two.

**Causal research question:** What is the causal effect of conception via ICSI compared to non-ICSI (conventional) IVF compared to natural conception on AEDC and NAPLAN outcomes for children?

**Exposure**: IVF with ICSI

**Primary outcome: *AEDC*** A score of <10^th^ centile in “2 or more” domains (Binary yes/no). ***NAPLAN***: Z score to be generated from our cohort using published mean and SD for national-scaled scores.

**Secondary outcomes:** ***AEDC*** A score in the lowest decile in each domain: 1) physical health and wellbeing, 2) social competence, 3) emotional maturity, 4) language and cognitive skills, and 5) communication skills and general knowledge. ***NAPLAN:*** Above or below minimum standard for each of the four domains (binary).

**Inclusion criteria:** Children born in the study time period.

**Exclusion criteria:** Multiple pregnancies, known congential anomaly.

*If there* ***ARE*** *differences in developmental and educational outcomes between ICSI and conventional IVF cohorts, we propose that this will be considered and reported as a primary finding since we have stated this sequential planned analysis a priori.*

The same methodology of causal inference, detailed in Part One and Two, will be employed for this analysis.

*Again, this analysis will focus on the* ***total effect*** *of ICSI conception adjusted for common cause confounders.*

**PART FOUR – OPEN, CLOSED and MEDIATED PATHS in the CAUSAL DIAGRAM / DAG**


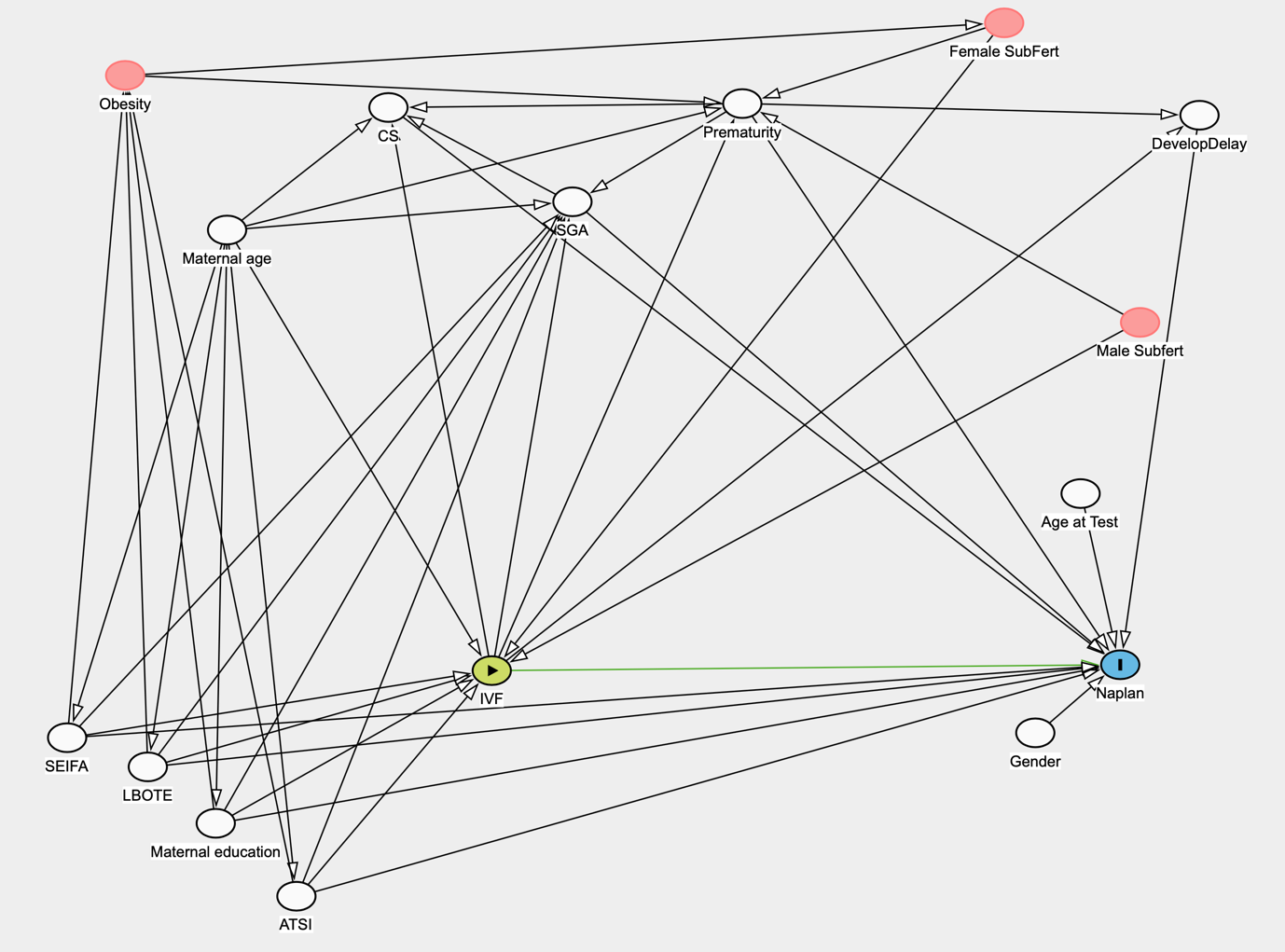


**Types of nodes (variables) in the above DAG**

**Common-cause confounders of the causal effect of the primary exposure (IVF) on the primary outcome (NAPLAN) [arrows from these variables to both IVF and NAPLAN]:** SEIFA, LBOTE, Maternal education, ATSI. Adjust all analyses (via IPW) for these as potential confounders.

**Mediators of the causal effect of** **the causal effect of the primary exposure (IVF) on the primary outcome (NAPLAN) [arrow from IVF to this variable, and then from this variable to NAPLAN, so they are “on the causal pathway”]:** CS, LBW, Prematurity (PTB), DevelopDelay.

**Variables that are common-cause confounders of the causal effect of the exposure (IVF) on one or more mediators:** Male SubFert and Female SubFert (confounds IVF and Prematurity), Maternal age (confounds IVF and all of CS, LBW and Prematurity).

**Variables that are common-cause confounders of mediators or causes of mediators (but are not mediators themselves):** Obesity, Maternal age.

**Mediators that common-cause confound the causal effect of another mediator on the outcome (NAPLAN) and are therefore “Exposure-Induced Mediator-Outcome Confounders” (EIMOC’s):** LBW (on Prematurity), Prematurity (on DevelopDelay).

**Proposed Causal Mediation Analyses**

*The relationships between variables represented in the DAG are complex, with mutltiple interacting pathways.*

Untangling this classification, we would be safe adjusting all analyses for **SEIFA**, **LBOTE**, **maternal** **education**, **ATSI**, **maternal age**, **male** and **female sub-fertility**, and **multiple pregnancy**. One could also adjust for obesity, but with adjustment for SEIFA, LBOTE, Maternal education, ATSI and female sub-fertility there are no remaining open back-door paths from exposure (IVF) to outcome (NAPLAN) that contain obesity.

That leaves four mediators: **caesarean Section (CS)**, **small for gestational Age (SGA)**, **prematurity (PTB)**, and **developemental delay**.

**Caesarean section** is effected by both prematurity and SGA thus is on the following mediated paths:

(1) IVF 🡪 SGA 🡪 🡪 CS 🡪 NAPLAN;

(2) IVF 🡪 Prematurity 🡪 C/S 🡪 NAPLAN; and

(3) IVF 🡪 C/S 🡪 NAPLAN.

**Proposed analysis:** Dependent on the total effect of IVF 🡪 C/S 🡪 NAPLAN (expected to be small) in our cohort we could consider **excluding** this mediator in the analysis.

**DevelopDelay** is on three mediated paths:

(1) IVF 🡪 SGA 🡪 Prematurity 🡪 DevelopDelay 🡪 NAPLAN;

(2) IVF 🡪 Prematurity 🡪 DevelopDelay 🡪 NAPLAN; and

(3) IVF 🡪 DevelopDelay 🡪 NAPLAN.

Even with all of these variables measured, these path-specific mediated effects cannot be estimated individually.

**Proposed analysis:** **excluding** DevelopDelay as a variable in the analysis allows its mediated effect to be absorbed through the other paths, that is, through the effects mediated by LBW and prematurity.

That leaves just **SGA** and **PTB** where the former often directly causes the latter.

**Proposed analysis:** Consider one or other of these mediators in a causal mediation analysis, or at least perform separate mediation analyses one mediator at a time. It would be possible to estimate the causal effect of IVF on NAPLAN mediated by Prematurity **not including** any effect through SGA and, in the same analysis, estimate the causal effect of IVF on NAPLAN mediated by SGA but it’s **not possible to separate** the causal effect of IVF on NAPLAN mediated effect SGA that **does** and **does not** go through **prematurity**, only the combined effect of the two mediated causal paths IVF 🡪 SGA 🡪 Prematurity 🡪 NAPLAN and IVF 🡪 SGA 🡪 NAPLAN.

*Consideration needs to be given to the heirachy of each of these mediators on the causal pathway as well as their effect on the remaining mediators.* ***We expect the proposed mediation analyses to be performed as a separate project.***
