# Supplementary material for: School-age outcomes among IVF-conceived children: A population-wide cohort study
Source: PLoS Med. 2023 Jan 24;20(1):e1004148. doi: 10.1371/journal.pmed.1004148 (PMC9873192; doi:10.1371/journal.pmed.1004148)
Supplement: S3 File — Tables A and B. Table A. Successful linkages by birth year. Table B. Annual cycle summaries from major Victorian IVF providers 2010–2014. (DOCX) [file pmed.1004148.s004.docx]

**Table A – Successful Linkages by Birth Year**

| **Baby birth year** | **IVF Births** | | | **Controls** | | |
| --- | --- | --- | --- | --- | --- | --- |
|  | Unlinked  N  (%) | Linked  N  (%) | Total | Unlinked  N  (%) | Linked  N  (%) | Total |
| 2005 | 68 | 834 | 902 | 6,755 | 52,053 | 58,808 |
|  | (7.54) | (92.46) |  | (11.49) | (88.51) |  |
| 2006 | 58 | 1,461 | 1,519 | 3,828 | 57,446 | 61,274 |
|  | (3.82) | (96.18) |  | (6.25) | (93.75) |  |
| 2007 | 96 | 1,572 | 1,668 | 6,096 | 57,018 | 63,114 |
|  | (5.76) | (94.24) |  | (9.66) | (90.34) |  |
| 2008 | 153 | 1,830 | 1,983 | 7,324 | 54,437 | 61,761 |
|  | (7.72) | (92.28) |  | (11.86) | (88.14) |  |
| 2009 | 81 | 1,612 | 1,693 | 5,150 | 57,497 | 62,647 |
|  | (4.78) | (95.22) |  | (8.22) | (91.78) |  |
| 2010 | 175 | 1,596 | 1,771 | 9,488 | 55,492 | 64,980 |
|  | (9.88) | (90.12) |  | (14.60) | (85.40) |  |
| 2011 | 1,280 | 237 | 1,517 | 48,594 | 10,194 | 58,788 |
|  | (84.38) | (15.62) |  | (82.66) | (17.34) |  |
| 2012 | 618 | 1,581 | 2,199 | 22,556 | 46,505 | 69,061 |
|  | (28.10) | (71.90) |  | (32.66) | (67.34) |  |
| 2013 | 2,066 | 336 | 2,402 | 57,848 | 11,011 | 68,859 |
|  | (86.01) | (13.99) |  | (84.01) | (15.99) |  |
| Total | 4,595 | 11,059 | 15,654 | 167,639 | 401,653 | 569,292 |
|  | (29.35) | (70.65) |  | (29.45) | (70.55) |  |

**Table B: Annual Cycle Summaries from Major Victorian IVF Providers 2010 - 2014 (Adapted from VARTA Annual reports^13,14^)**

|  | Number of women treated | Age at first treatment | | | Number of cycles | Number of ICSI* cycles | Number of thaw cycles | Number of births |
| --- | --- | --- | --- | --- | --- | --- | --- | --- |
|  |  | <35 | 35-39 | >39 |  |  |  |  |
| **2008/2009** | | | | | | | | |
| City Fertility Centre | 340 | 125 | 121 | 94 | 287 | 151 | 183 | 66 |
| Melbourne IVF | 2549 | 756 | 1058 | 735 | 2089 | 1114 | 1266 | 432 |
| Monash IVF | 3012 | 1619 | 1326 | 1900 | 2831 | 1953 | 777 | 927 |
| Reprod. Services, Royal Women’s Hospital (Melbourne IVF) | 1976 | 752 | 709 | 515 | 1469 | 777 | 1017 | 252 |
| Repromed Mildura | 68 | 39 | 22 | 7 | 57 | 38 | 22 | 13 |
| Ballarat IVF | 287 | 135 | 103 | 49 | 206 | 134 | 140 | 66 |
| Total (included in current study) | 7877 | 3252 | 3214 | 3244 | 6676 | 3995 | 3243 | 1677 |
| Total (Victoria) | 8232 | 3426 | 3339 | 3300 | 6939 | 4167 | 3405 | 1756 |
| % cycles included | 95.7 | 94.9 | 96.3 | 98.3 | 96.2 | 95.9 | 95.2 | 95.5 |
| **2012/2014** | | | | | | | | |
| City Fertility Centre | 711 | 270 | 253 | 188 | 530 | 313 | 346 | 88 |
| Melbourne IVF | 4030 | 1464 | 1456 | 1110 | 2925 | 2119 | 1922 | 600 |
| Monash IVF | 4785 | 1702 | 1731 | 1352 | 3651 | 2697 | 2133 | 740 |
| Reprod. Services, Royal Women’s Hospital (Melbourne IVF) | 1163 | 510 | 377 | 276 | 883 | 584 | 439 | 152 |
| Repromed Mildura | Ceased 2012 | | | | | | | |
| Ballarat IVF | 323 | 164 | 104 | 55 | 206 | 140 | 153 | 109 |
| Total (included in current study) | 10689 | 3946 | 3817 | 2926 | 7989 | 5713 | 4840 | 1580 |
| Total (Victoria) | 11012 | 4110 | 3921 | 2981 | 8195 | 5853 | 4993 | 1689 |
| % cycles included | 97.1 | 96.0 | 97.3 | 98.2 | 97.5 | 97.6 | 96.9 | 93.5 |

*ICSI = Intra-cytoplasmic sperm injection
