# Supplementary figures and images for: School-age outcomes among IVF-conceived children: A population-wide cohort study
Source: PLoS Med. 2023 Jan 24;20(1):e1004148. doi: 10.1371/journal.pmed.1004148 (PMC9873192; doi:10.1371/journal.pmed.1004148)

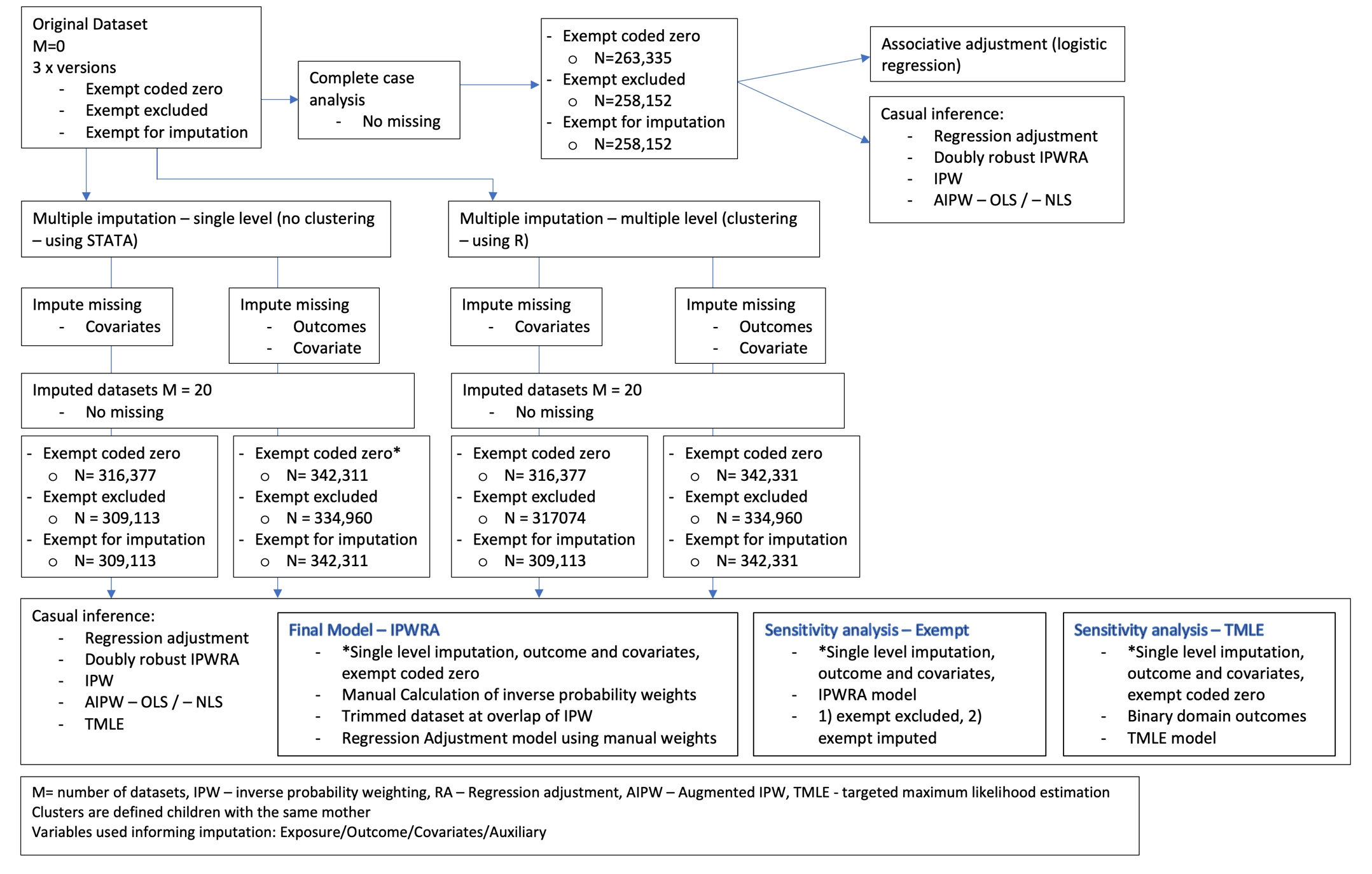


Fig A – Analysis Flow Chart (National Assessment Program – Literacy and Numeracy, NAPLAN)

Supplement: S4 File — Fig A. Analysis flow chart (NAPLAN). (DOCX) [file pmed.1004148.s005.docx]
