# Supplementary material for: School-age outcomes among IVF-conceived children: A population-wide cohort study
Source: PLoS Med. 2023 Jan 24;20(1):e1004148. doi: 10.1371/journal.pmed.1004148 (PMC9873192; doi:10.1371/journal.pmed.1004148)

**Multiple imputation dataset details (National Assessment Program – Literacy and Numeracy, NAPLAN)**

Observations:

Complete 263,335

Incomplete 78,976 (M = 20 imputations)

________________

Total 342,311

Variables:

Imputed: #16; (# missing) L1_napscal1z(17886) L1_napscal2z(18391) L1_napscal3z(17728) L1_napscal4z(17885) L1_napscal5z(18919) L1_seifa5(339) L2_P1schooled_cluster(6422) L2_P1nonschooled_cluster(10616)

L2_P2schooled(47142) L2_P2nonschooled(52635) L2_parity(220) expParity(220)

Passive: #1; (# missing) outcome(25934)

Regular: #6; (# missing) L1_testyear(0) L2_lbote(0) L1_gender(0) L1_matage(0) L1_testyear(0) L2_lbote(0)

Auxiliary Variables L1_atsi L1_matage Aux_L1_remote Aux_L1_P1origin

**Multiple imputation model (National Assessment Program – Literacy and Numeracy, NAPLAN) – STATA code**

mi impute chained ///

(pmm,knn(3)) L1_bwc L1_gest L2_parity testyearsq exptestyear expParity gendertestyear ///

(mlogit) L1_seifa5 L1_mob L2_P1schooled L2_P1postschool = exposure L1_gender ///

L1_lbote L1_atsi L1_matage Aux_L1_remote Aux_L1_P1origin outcome L1_phys L1_soc L1_emot L1_lang L1_comgen expmatage///

,chainonly burnin(100) savetrace(impstats,replace) rseed(1234)

**Table A: Missing data summary (National Assessment Program – Literacy and Numeracy, NAPLAN)**

|  | | | **Non-Missing Data** | | | |
| --- | --- | --- | --- | --- | --- | --- |
| **Variable** | **No. of Missing Observations** | **No. Observed** | **Unique values** | **Range of Values** | | |
|  |  |  |  | **Minimum** | **Maximum** | |
| Grammar and Punctuation (z-score) | 17,886 | 324,425 | 460 | -5.84 | | 5.61 |
| Numeracy (z-score) | 18,391 | 323,920 | >500 | -6.52 | | 5.29 |
| Reading (z-score) | 17,728 | 324,583 | >500 | -5.67 | | 6.73 |
| Spelling (z-score) | 17,885 | 324,426 | >500 | -3.51 | | 3.03 |
| Writing (z-score) | 18,919 | 323,392 | 295 | -6.10 | | 5.56 |
| SEIFA | 339 | 341,972 | 5 | 1 | | 5 |
| Maternal School Education | 6,422 | 335,889 | 4 | 0 | | 3 |
| Maternal Post School Education | 10,616 | 331,695 | 4 | 0 | | 3 |
| Second Parent School Education | 47,142 | 295,169 | 4 | 0 | | 3 |
| Second Parent Post School Education | 52,635 | 289,676 | 4 | 0 | | 3 |
| Parity | 220 | 342,091 | 6 | 0 | | >6 |
| Outcome (combined Z-score) | 25,934 | 316,377 | >500 | -4.58 | | 3.50 |

**Missing data nested summary**

1. expParity(220) <-> L2_parity(220)

2. L1_seifa5(339)

3. L2_P1schooled_cluster(6422) <-> L2_P1schooled(6422)

4. L1_P1occ(7425)

5. L2_P1nonschooled_cluster(10616) <-> L2_P1nonschooled(10616) -> L1_P1ed_qualif(10914)

6. L1_napscal3z(17728) -> outcome_preimp(25934) <-> outcome(25934) <->

MIL1_napscalztotal(25934) <-> L1_napscalztotal(25934) <-> L1_napscalav(25934)

7. L1_napscal4z(17885) -> L1_napscal1z(17886) -> outcome_preimp(25934) <-> outcome(25934)

<-> MIL1_napscalztotal(25934) <-> L1_napscalztotal(25934) <-> L1_napscalav(25934)

8. L1_napscal2z(18391) -> outcome_preimp(25934) <-> outcome(25934) <->

MIL1_napscalztotal(25934) <-> L1_napscalztotal(25934) <-> L1_napscalav(25934)

9. L1_napscal5z(18919) -> outcome_preimp(25934) <-> outcome(25934) <->

MIL1_napscalztotal(25934) <-> L1_napscalztotal(25934) <-> L1_napscalav(25934)

10. L1_P2occ(44071)

11. L2_P2schooled(47142)

12. L2_P2nonschooled(52635)

**Fig A: NAPLAN (National Assessment Program – Literacy and Numeracy) convergence**


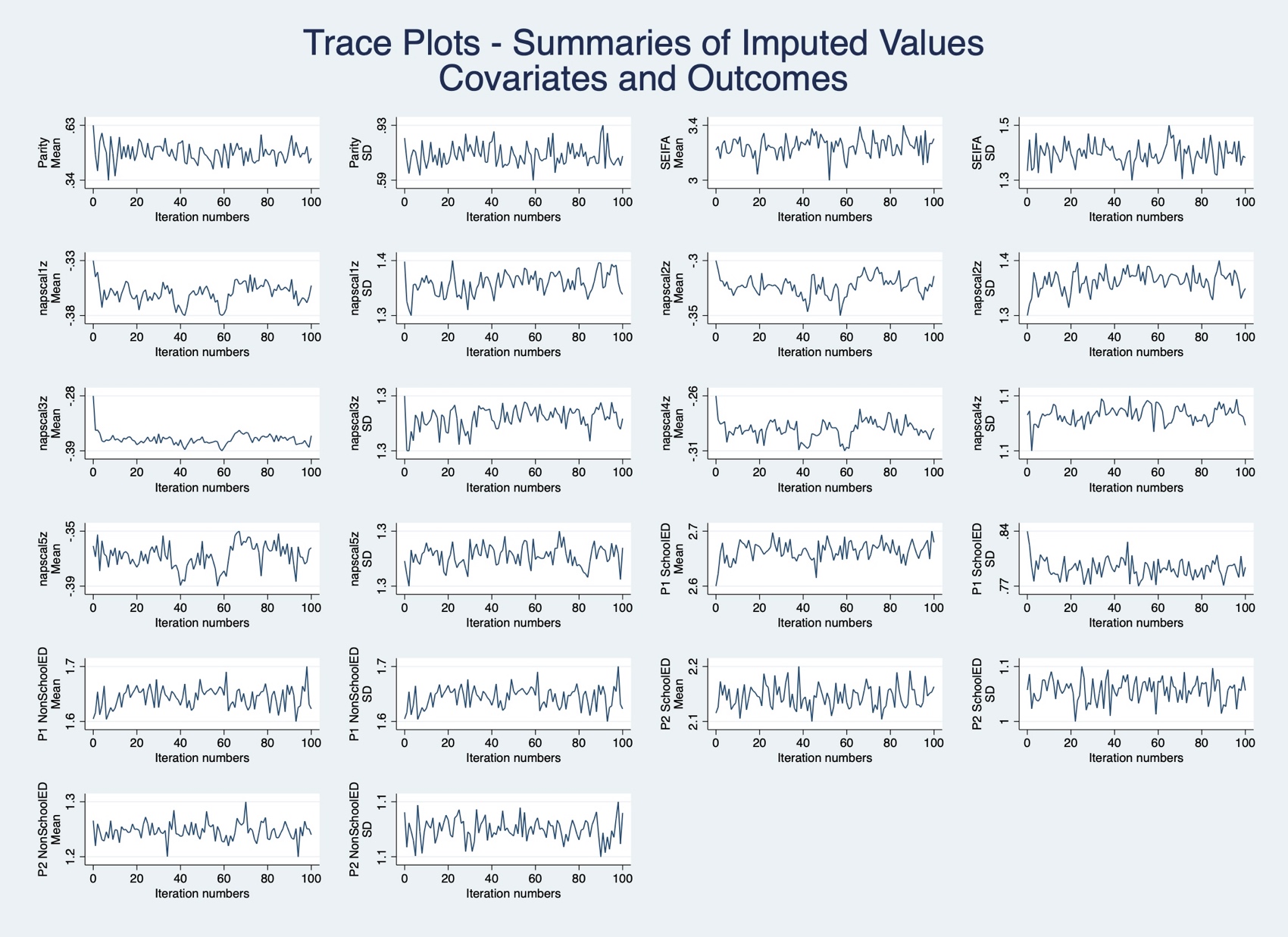


**Fig B: NAPLAN (National Assessment Program – Literacy and Numeracy) density plots of observed and imputed data**


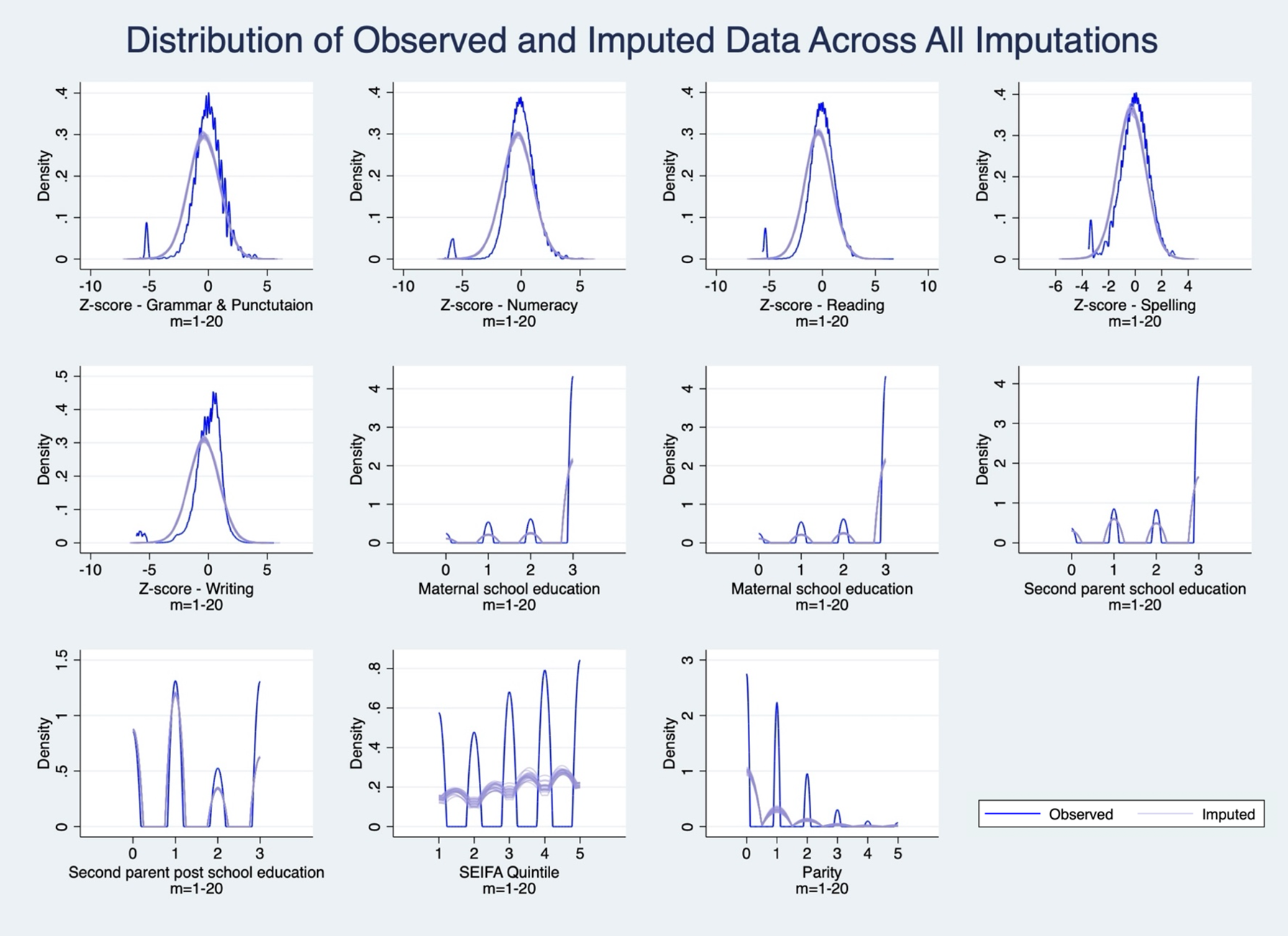


**Fig C: NAPLAN (National Assessment Program – Literacy and Numeracy) distribution of outcome and covariates after imputation in m=1 dataset**


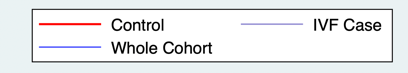


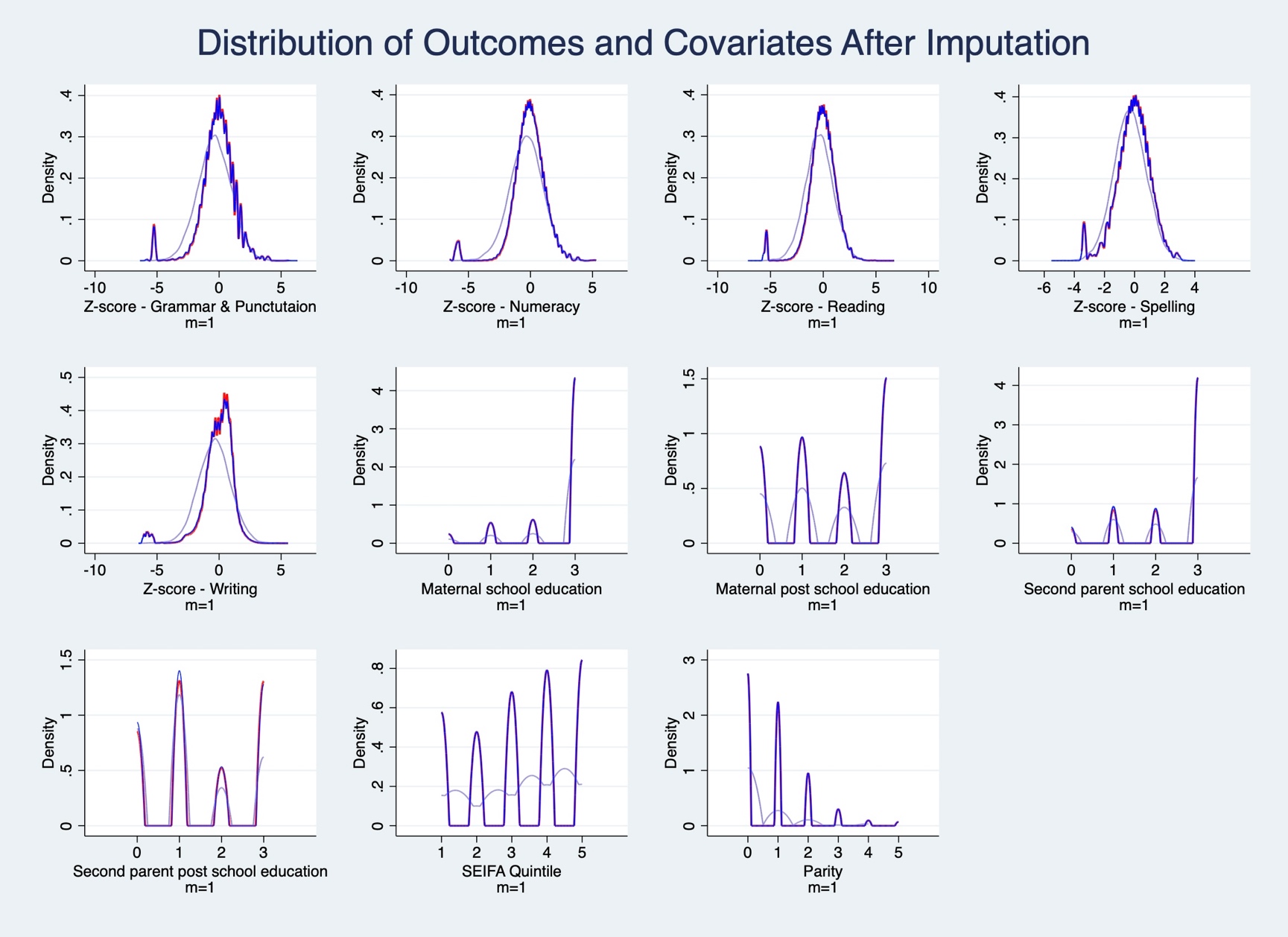

Supplement: S5 File — Table A. Missing data summary (NAPLAN). Fig A. NAPLAN convergence. Fig B. NAPLAN density plots of observed and imputed data. Fig C. NAPLAN distribution of outcome and covariates after imputation in m = 1 dataset. (DOCX) [file pmed.1004148.s006.docx]
