# Supplementary material for: School-age outcomes among IVF-conceived children: A population-wide cohort study
Source: PLoS Med. 2023 Jan 24;20(1):e1004148. doi: 10.1371/journal.pmed.1004148 (PMC9873192; doi:10.1371/journal.pmed.1004148)

**Fig A: AEDC (Australian Early Development Census) Imputation #1**

A B C

**
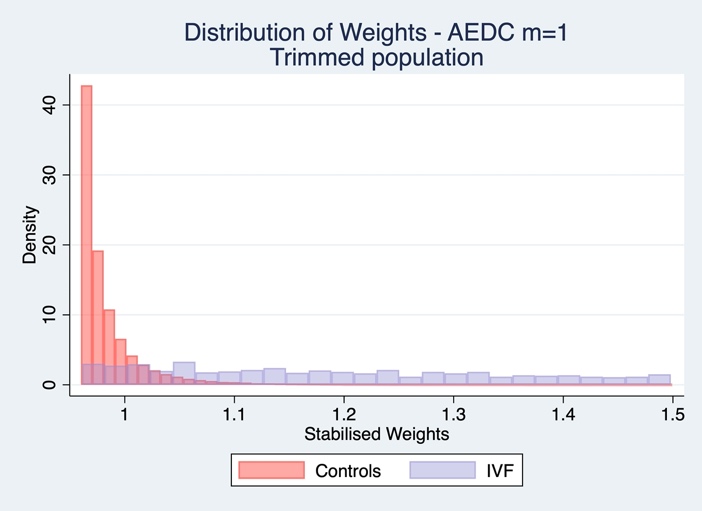

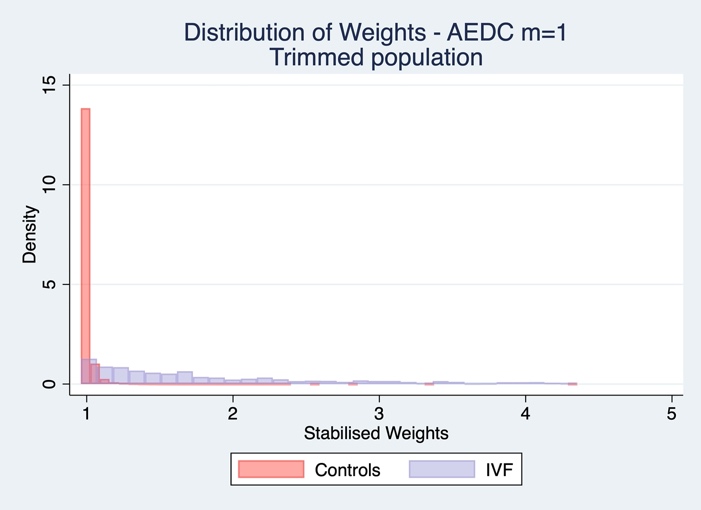

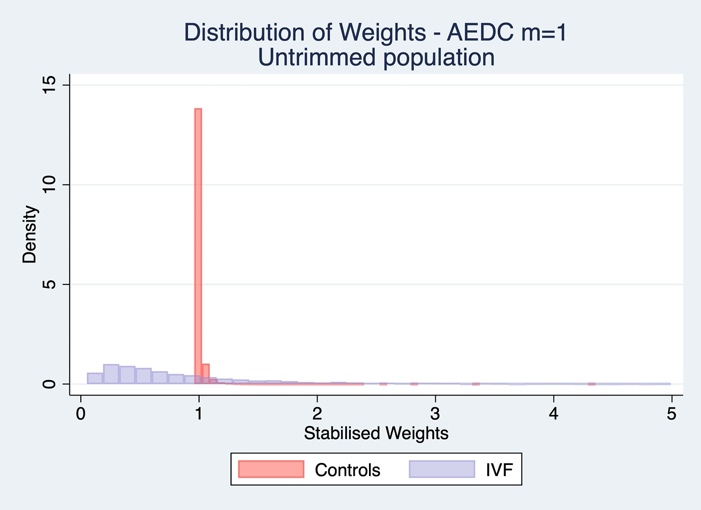
**

**Fig B: AEDC (Australian Early Development Census) Imputation #13**

A B C


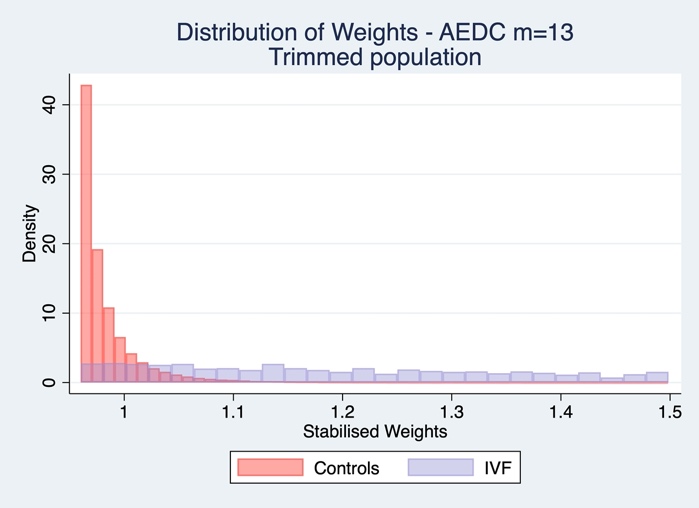

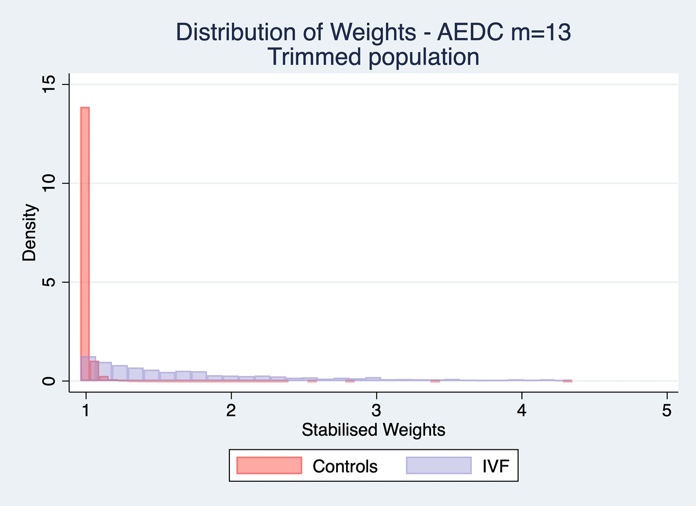

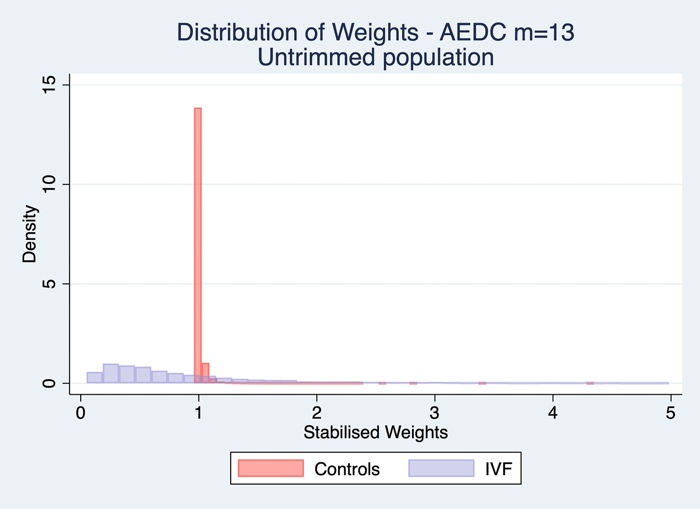


**Fig C: NAPLAN (National Assessment Program – Literacy and Numeracy) Imputation #1**

A B C

**
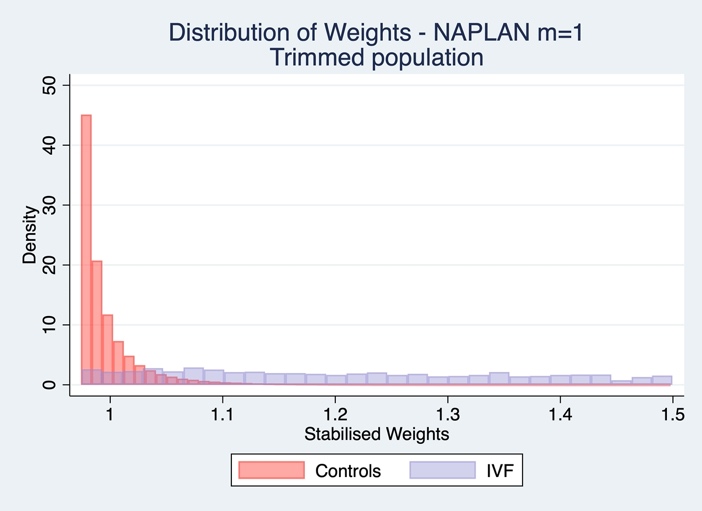

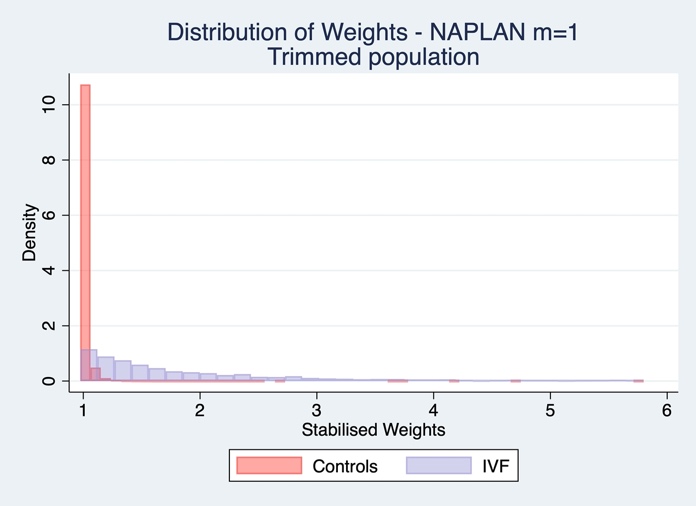

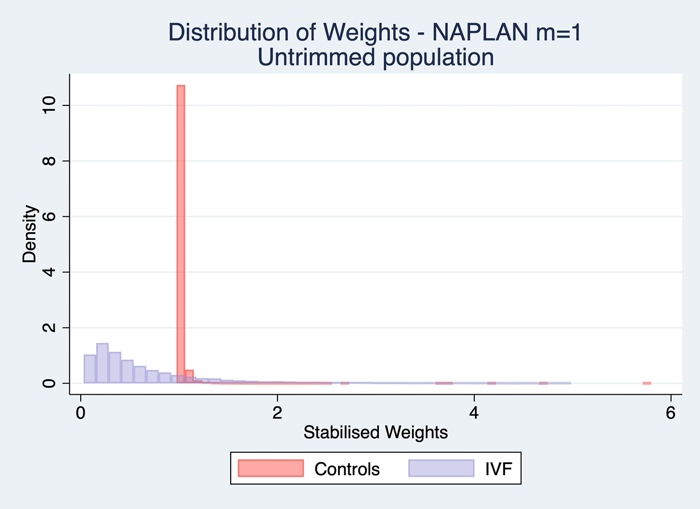
**

**Fig D: NAPLAN (National Assessment Program – Literacy and Numeracy) Imputation #13**

A B C


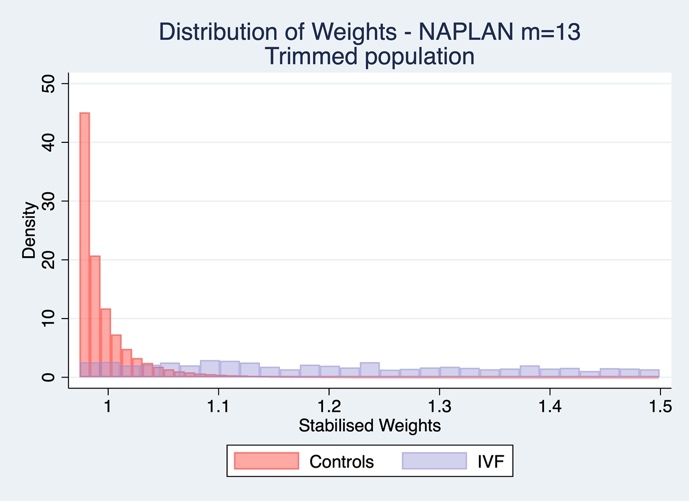

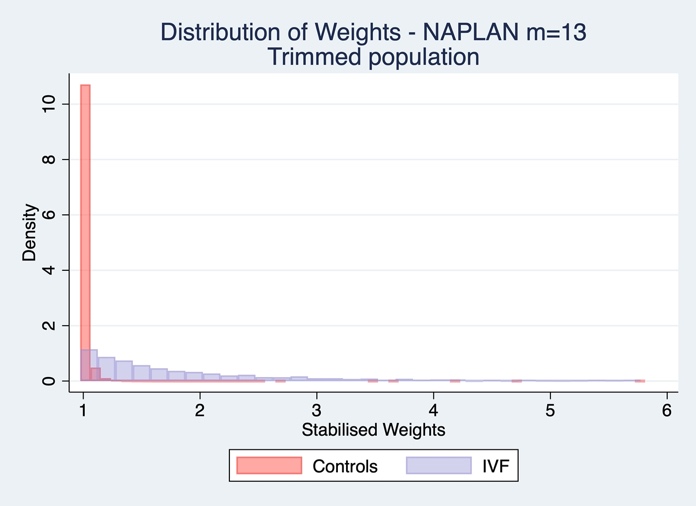

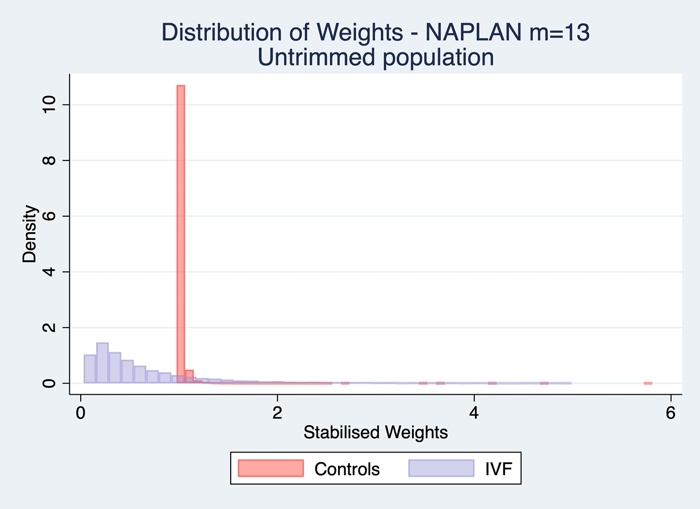

Supplement: S6 File — Figs A–D: Distribution and overlap of manually calculated stabilised weights. Fig A. AEDC imputation #1. Fig B. AEDC imputation #13. Fig C. NAPLAN imputation #1. Fig D. NAPLAN imputation #13. (DOCX) [file pmed.1004148.s007.docx]
