# Supplementary material for: School-age outcomes among IVF-conceived children: A population-wide cohort study
Source: PLoS Med. 2023 Jan 24;20(1):e1004148. doi: 10.1371/journal.pmed.1004148 (PMC9873192; doi:10.1371/journal.pmed.1004148)
Supplement: S7 File — Figs A–C: Variable standardised mean differences. Fig A. NAPLAN imputation #1 variable standardised mean differences. Fig B. NAPLAN imputation #13 variable standardised mean differences. Fig C. AEDC imputation #7 variable standardised mean differences. (DOCX) [file pmed.1004148.s008.docx]

**Fig A: NAPLAN (National Assessment Program – Literacy and Numeracy) Imputation #1 Variable Standardised Mean Differences**

**A
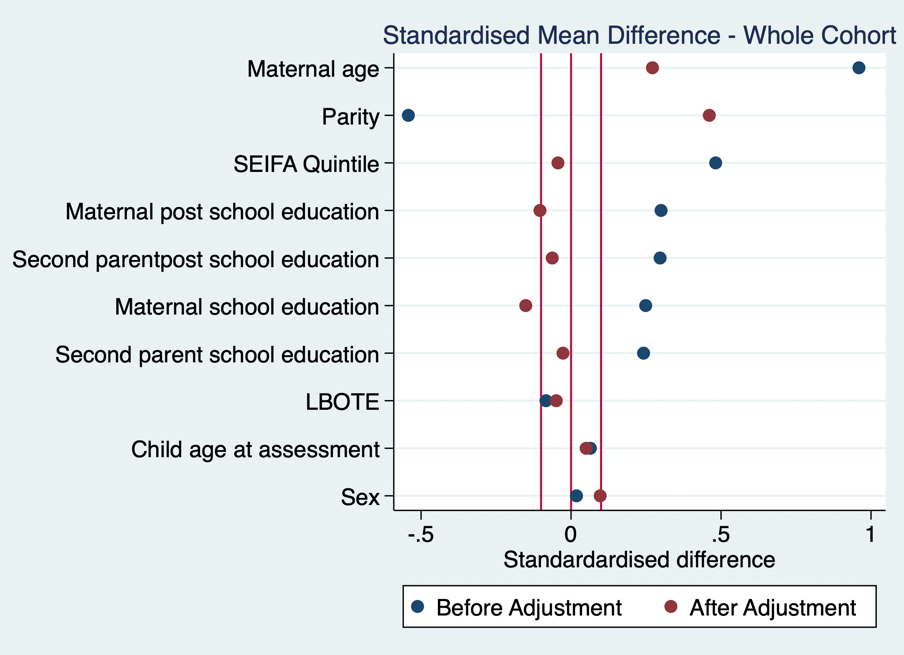
 B**
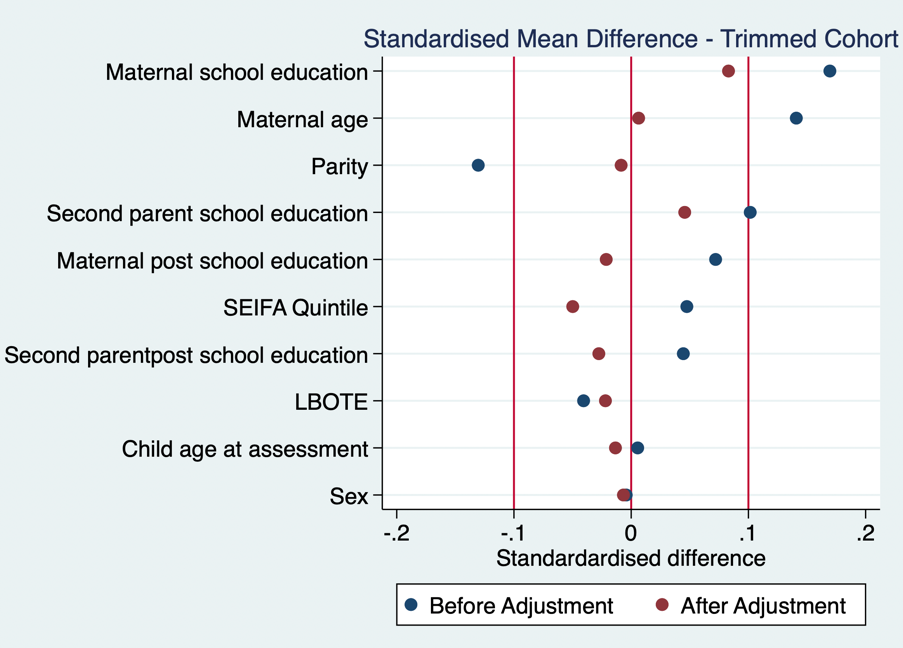


Fig A-C – examples of standardised mean differences in imputed datasets after adjustment with stabilised weights (A) and after trimming of dataset to ensure overlap of weights in cases and control populations (B)

Abbreviations: LBOTE – language background other than English, SEIFA – Socioeconomic index for areas (relative deprivation based on post-code)

**Fig B: NAPLAN (National Assessment Program – Literacy and Numeracy) Imputation #13 Variable Standardised Mean Differences**

**A
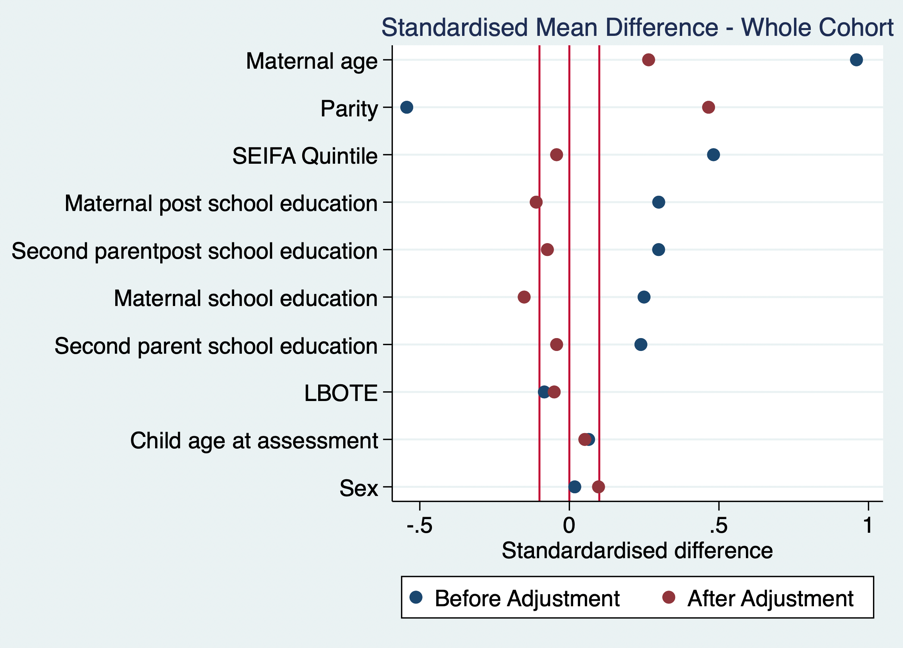
 B**
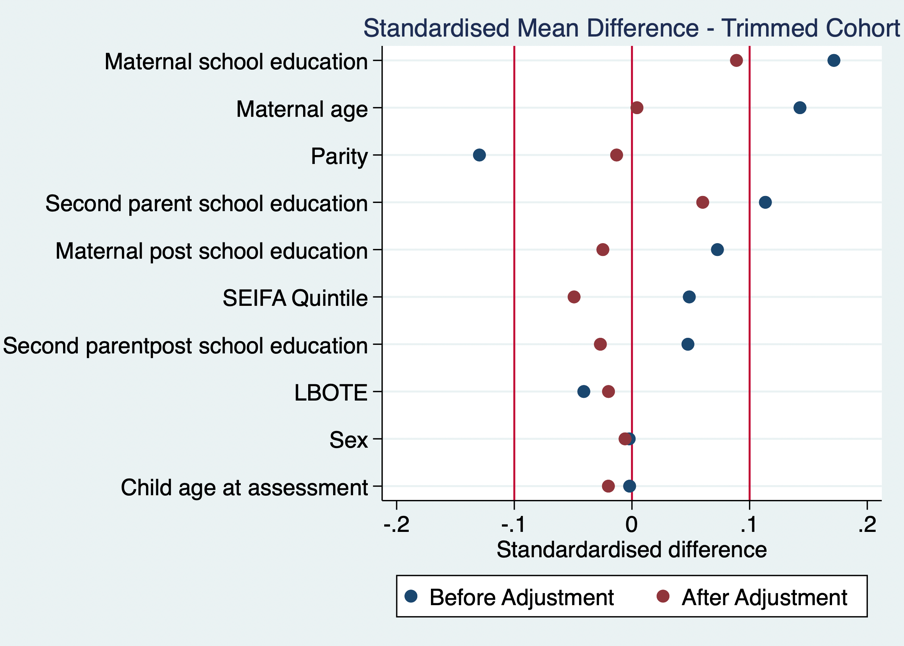


Fig A-C – examples of standardised mean differences in imputed datasets after adjustment with stabilised weights (A) and after trimming of dataset to ensure overlap of weights in cases and control populations (B)

Abbreviations: LBOTE – language background other than English, SEIFA – Socioeconomic index for areas (relative deprivation based on post-code)

**Fig C: AEDC (Australian Early Development Census) Imputation #7 Variable Standardised Mean Differences**

**A
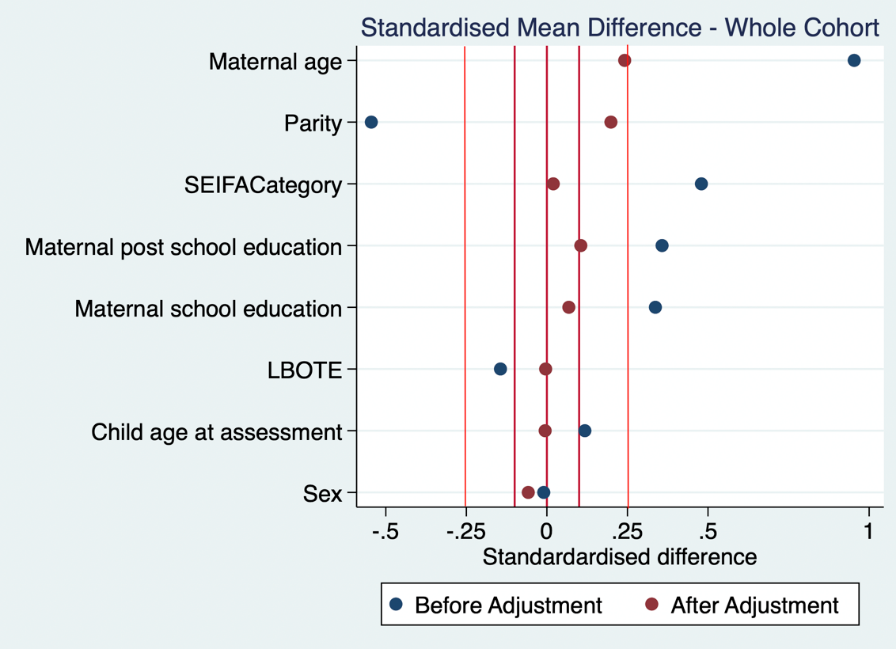
 B
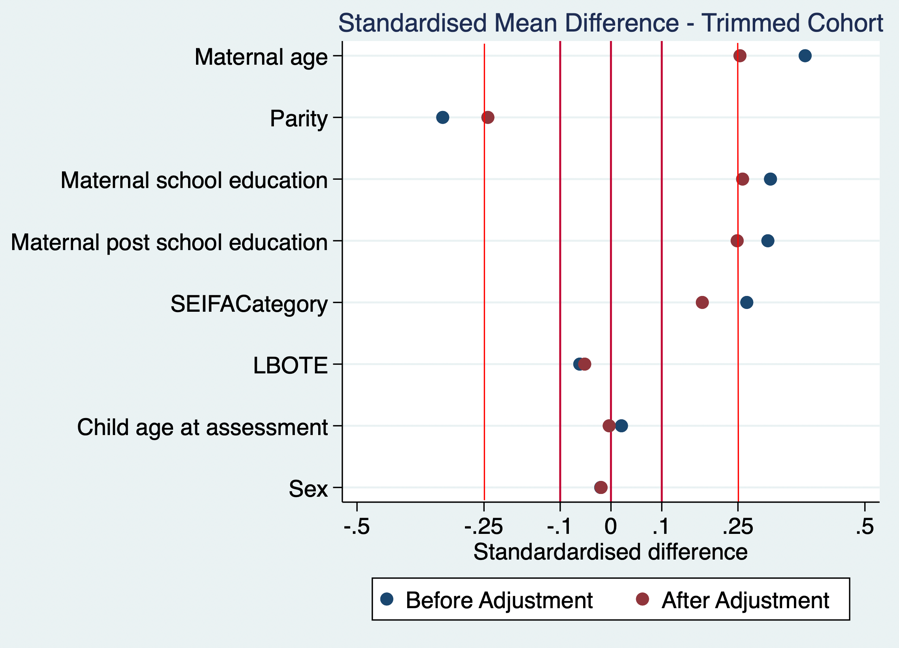
**

Figs A-C – examples of standardised mean differences in imputed datasets after adjustment with stabilised weights (A) and after trimming of dataset to ensure overlap of weights in cases and control populations (B)
