# Supplementary material for: School-age outcomes among IVF-conceived children: A population-wide cohort study
Source: PLoS Med. 2023 Jan 24;20(1):e1004148. doi: 10.1371/journal.pmed.1004148 (PMC9873192; doi:10.1371/journal.pmed.1004148)
Supplement: S8 File — Tables A and B. Table A. Sensitivity analysis–AEDC (special needs multiply imputed). Table B. Sensitivity analysis–AEDC (special needs excluded). (DOCX) [file pmed.1004148.s009.docx]

**Table A – Sensitivity Analysis – AEDC (Australian Early Development Census): special needs multiply imputed**

|  | Non-imputed crude data | |  | Imputed data – causal model^a^ | | | | |
| --- | --- | --- | --- | --- | --- | --- | --- | --- |
|  | Proportions | |  | Predicted Proportions | |  | Regression Co-efficient: | |
|  | Controls  **(n=168,503)** | IVF  **(n=4,697)** |  | Controls  **(n=168,503)** | IVF  **(n=,485 -1,541^b^)** |  | ATE Risk difference  (95% Confidence Interval) | ATE Risk Ratio  (95% Confidence Interval) |
| **Primary outcome (developmentally vulnerable in 2 or more domains)** | | | | | | | | |
|  | 0.092 | 0.054 |  | 0.093 | 0.075 | | -0.018  (-0.044 to 0.009) | 0.81  (0.53 to 1.24) |
| **Secondary outcomes (developmentally vulnerable across individual domains)** | | | | | | |  |  |
| Physical Health and Wellbeing | 0.077 | 0.056 |  | 0.059 | 0.077 | | -0.018  (-0.043 to 0.006) | 0.76  (0.50 to 1.17) |
| Social Competence | 0.082 | 0.056 |  | 0.083 | 0.073 | | -0.009  (-0.036 to 0.017) | 0.88  (0.58 to 1.36) |
| Emotional Maturity | 0.076 | 0.054 |  | 0.077 | 0.053 | | -0.024  (-0.047 to -0.002)^c^ | 0.68  (0.45 to 1.05) |
| Language and Cognitive Skills (school based) | 0.057 | 0.025 |  | 0.057 | 0.054 | | -0.003  (-0.027 to 0.020) | 0.94  (0.61 to 1.44) |
| Communication and General Knowledge | 0.065 | 0.030 |  | 0.065 | 0.046 | | -0.019  (-0.040 to -0.003)^c^ | 0.71  (0.46 to 1.09) |
| Children with missing outcome data (5.2%) identified as having special needs are included – their outcome category has been multiply imputed.  a: Causal Model: imputed data pooled estimates – regression adjustment model with stabilised inverse probability weighting, trimmed for complete weight overlap.  Abbreviations: AEDC – Australian Early Development Census, IVF – in-vitro fertilisation (cases), ATE - Average Treatment Effect  b: small variation in case number for each of the 20 imputation datasets  c: 95% confidence interval does not cross the null | | | | | | | | |

**Table B – Sensitivity Analysis – AEDC (Australian Early Development Census): special needs excluded**

|  | Non-imputed crude data | |  | Imputed data – causal model^a^ | | | | |
| --- | --- | --- | --- | --- | --- | --- | --- | --- |
|  | Proportions | |  | Predicted Proportions | |  | Regression Co-efficient: | |
|  | Controls  **(n=159,081)** | IVF  **(n=4,441)** |  | Controls  **(n=158,286)** | IVF  **(1,426 -1,469^b^)** |  | ATE Risk difference  (95% Confidence Interval) | ATE Risk Ratio  (95% Confidence Interval) |
| **Primary outcome (developmentally vulnerable in 2 or more domains)** | | | | | | | | |
|  | 0.092 | 0.054 |  | 0.091 | 0.075 | | -0.015  (-0.042 to 0.012) | 0.83  (0.53 to 1.31) |
| **Secondary outcomes (developmentally vulnerable across individual domains)** | | | | | | |  |  |
| Physical Health and Wellbeing | 0.077 | 0.056 |  | 0.076 | 0.059 | | -0.017  (-0.042 to 0.008) | 0.77  (0.49 to 1.22) |
| Social Competence | 0.082 | 0.055 |  | 0.081 | 0.075 | | -0.006  (-0.033 to 0.021) | 0.92  (0.59 to 1.45) |
| Emotional Maturity | 0.076 | 0.053 |  | 0.076 | 0.052 | | -0.024  (-0.046 to -0.003)^c^ | 0.68  (0.43 to 1.07) |
| Language and Cognitive Skills (school based) | 0.057 | 0.025 |  | 0.056 | 0.053 | | -0.003  (-0.026 to 0.020) | 0.94  (0.60 to 1.49) |
| Communication and General Knowledge | 0.065 | 0.030 |  | 0.063 | 0.049 | | -0.015  (-0.037 to 0.008) | 0.77  (0.49 to 1.21) |
| Children with missing outcome data (5.2%) identified as having special needs are excluded  a: Causal Model: imputed data pooled estimates – regression adjustment model with stabilised inverse probability weighting, trimmed for complete weight overlap.  Abbreviations: AEDC – Australian Early Development Census, IVF – in-vitro fertilisation (cases), ATE - Average Treatment Effect  b: small variation in case number for each of the 20 imputation datasets | | | | | | | | |

c: 95% confidence interval does not cross the null
