# Supplementary material for: School-age outcomes among IVF-conceived children: A population-wide cohort study
Source: PLoS Med. 2023 Jan 24;20(1):e1004148. doi: 10.1371/journal.pmed.1004148 (PMC9873192; doi:10.1371/journal.pmed.1004148)
Supplement: S9 File — Tables A and B. Table A. Sensitivity analysis–NAPLAN (exempt multiply imputed). Table B. Sensitivity analysis–NAPLAN (exempt excluded). (DOCX) [file pmed.1004148.s010.docx]

**Table A – Sensitivity Analysis – National Assessment Program – Literacy and Numeracy (NAPLAN): exempt multiply imputed**

|  | Non-imputed crude data | |  | Imputed data – causal model^a^ | | | |
| --- | --- | --- | --- | --- | --- | --- | --- |
|  | Mean (SE) | |  | Potential Outcome  Mean | |  | Regression co-efficient:  ATE mean difference  (95% Confidence Interval) |
|  | Control  **(n=333,335)** | IVF  **(n=8976)** |  | Control  **(n=333,335)** | IVF  **(n=1986-2016^b^)** |  |  |
| **Primary outcome - Overall Z-score** | | |  | | | | |
|  | -0.007 (0.002) | 0.265 (0.011) |  | -0.027 | 0.023 | | 0.004 (-0.046 to 0.053) |
| **Secondary outcomes – Individual domains Z-score** | | |  | | | | |
| Grammar and Punctuation | -0.027 (0.002) | 0.304 (0.013) |  | -0.001 | -0.007 | | -0.006 (-0.060 to 0.049) |
| Numeracy | -0.031 (0.002) | 0.274 (0.012) |  | 0.008 | 0.022 | | 0.014 (-0.038 to 0.066) |
| Reading | -0.027 (0.002) | 0.325 (0.012) |  | 0.003 | -0.007 | | -0.010 (-0.063 to 0.043) |
| Spelling | -0.003 (0.002) | 0.185 (0.012) |  | -0.031 | -0.022 | | -0.010 (-0.061 to 0.042) |
| Writing | -0.028 (0.002) | 0.238 (0.011) |  | 0.001 | 0.032 | | 0.031 (-0.020 to 0.081) |
| Children with missing outcome data exempt from NAPLAN (2.1%) are included – with their missing z-score multiply imputed.  a: Causal Model: imputed data pooled estimates – Regression adjustment model with stabilised inverse probability weighting, trimmed for complete weight overlap  Abbreviations: NAPLAN – National Assessment Program for Literacy and Numeracy, IVF – in-vitro fertilisation (cases), ATE - Average Treatment Effect, SE – standard error  b: small variation in case number for each of the 20 imputation datasets | | | | | | | |

**Table B – Sensitivity Analysis – National Assessment Program – Literacy and Numeracy (NAPLAN): exempt excluded**

|  | Non-imputed crude data | |  | Imputed data – causal model^a^ | | | |
| --- | --- | --- | --- | --- | --- | --- | --- |
|  | Mean (SE) | |  | Potential Outcome  Mean (SE) | |  | Regression co-efficient:  ATE mean difference  (95% Confidence Interval) |
|  | Control  **(n=326,117)** | IVF  **(n=8,843)** |  | Control  **(n=326,117)** | IVF  **(n=1953-1976^b^)** |  |  |
| **Primary outcome - Overall Z-score** | | |  | | | | |
|  | -0.007 (0.002) | 0.265 (0.011) |  | -0.022 | 0.020 | | 0.002 (-0.047 to 0.051) |
| **Secondary outcomes – Individual domains Z-score** | | |  | | | | |
| Grammar and Punctuation | 0.012 (0.002) | 0.292 (0.012) |  | 0.006 | -0.001 | | -0.007 (-0.061 to 0.048) |
| Numeracy | 0.018 (0.002) | 0.265 (0.012) |  | 0.013 | 0.023 | | 0.010 (-0.042 to 0.062) |
| Reading | 0.015 (0.002) | 0.314 (0.012) |  | 0.009 | -0.003 | | -0.012 (-0.065 to 0.041) |
| Spelling | -0.010 (0.002) | 0.176 (0.011) |  | -0.017 | -0.028 | | -0.011 (-0.063 to 0.040) |
| Writing | 0.016 (0.002) | 0.228 (0.011) |  | 0.007 | 0.039 | | 0.032 (-0.018 to 0.082) |
| Children with missing outcome data exempt from NAPLAN (2.1%) are excluded.  a: Causal Model: imputed data pooled estimates – Regression adjustment model with stabilised inverse probability weighting, trimmed for complete weight overlap  Abbreviations: NAPLAN – National Assessment Program for Literacy and Numeracy, IVF – in-vitro fertilisation (cases), ATE - Average Treatment Effect, SE – standard error  b: small variation in case number for each of the 20 imputation datasets | | | | | | | |
