# Supplementary material for: School-age outcomes among IVF-conceived children: A population-wide cohort study
Source: PLoS Med. 2023 Jan 24;20(1):e1004148. doi: 10.1371/journal.pmed.1004148 (PMC9873192; doi:10.1371/journal.pmed.1004148)
Supplement: S10 File — Table A–Sensitivity analysis–binary outcomes TMLE. (DOCX) [file pmed.1004148.s011.docx]

**Table A – Sensitivity Analysis – Binary Outcomes, Targeted maximum likelihood estimation (TMLE)**

|  | Non-imputed crude data | |  | Imputed data – causal model^a^ | | | | | | |  |
| --- | --- | --- | --- | --- | --- | --- | --- | --- | --- | --- | --- |
|  | Proportions | |  | Predicted  Proportions | |  | | Regression co-efficient: | | |  |
|  | Control | IVF |  | Control | IVF | |  | | ATE Risk difference  (95% Confidence Interval) | ATE Risk Ratio  (95% Confidence Interval) | |
| **AEDC** | **(n=168,503)** | **(n=4,697)** |  | **(n=173,200)** | **(n=173,200)** | | | | | | |
| **Primary Outcome – Developmentally vulnerable in two or more AEDC domains** | | | | | | | | | | |  |
|  | 0.140 | 0.104 |  | 0.139 | 0.121 | | | | -0.017  (-0.023 to -0.011) | 0.86  (0.82 to 0.91) | |
| **NAPLAN** | **(n=333,335** | **(n=8976)** |  | **(n=342,311** | **(n=342,311)** | | | |  |  | |
| **Domains below ‘National Minimum Standard’** | | |  |  |  | | | |  |  | |
| Grammar and Punctuation | 0.051 | 0.029 |  | 0.052 | 0.042 | | | | -0.011  (-0.013 to -0.008) | 0.84  (0.75 to 0.84) | |
| Numeracy | 0.0382 | 0.0236 |  | 0.039 | 0.031 | | | | -0.008  (-0.010 to -0.006) | 0.85  (0.74 to 0.85) | |
| Reading | 0.0444 | 0.0260 |  | 0.048 | 0.037 | | | | -0.009  (-0.012 to -0.007) | 0.83  (0.75 to 0.85) | |
| Spelling | 0.0494 | 0.0275 |  | 0.051 | 0.041 | | | | -0.010  (-0.012 to -0.007) | 0.81  (0.76 to 0.85) | |
| Writing | 0.0326 | 0.0202 |  | 0.034 | 0.025 | | | | -0.008  (-0.010 to -0.006) | 0.78  (0.69 to 0.81) | |

a: Targeted maximum likelihood estimation (TMLE) causal model, default option - tmle (TMLE algorithm plus super-Learner ensemble):
Imputed data pooled estimates

Abbreviations: NAPLAN – National Assessment Program for Literacy and Numeracy, AEDC – Australian Early Development Census, IVF – in-vitro fertilisation (cases),
ATE - Average Treatment Effect
