# Supplementary material for: School-age outcomes among IVF-conceived children: A population-wide cohort study
Source: PLoS Med. 2023 Jan 24;20(1):e1004148. doi: 10.1371/journal.pmed.1004148 (PMC9873192; doi:10.1371/journal.pmed.1004148)
Supplement: S11 File — Figs A and B. Fig A. Sensitivity analysis AEDC primary outcome–E-value estimation. Fig B. Sensitivity analysis NAPLAN primary outcome–E-value estimation. (DOCX) [file pmed.1004148.s012.docx]

**Fig A – Sensitivity Analysis AEDC Primary Outcome – E-value Estimation**


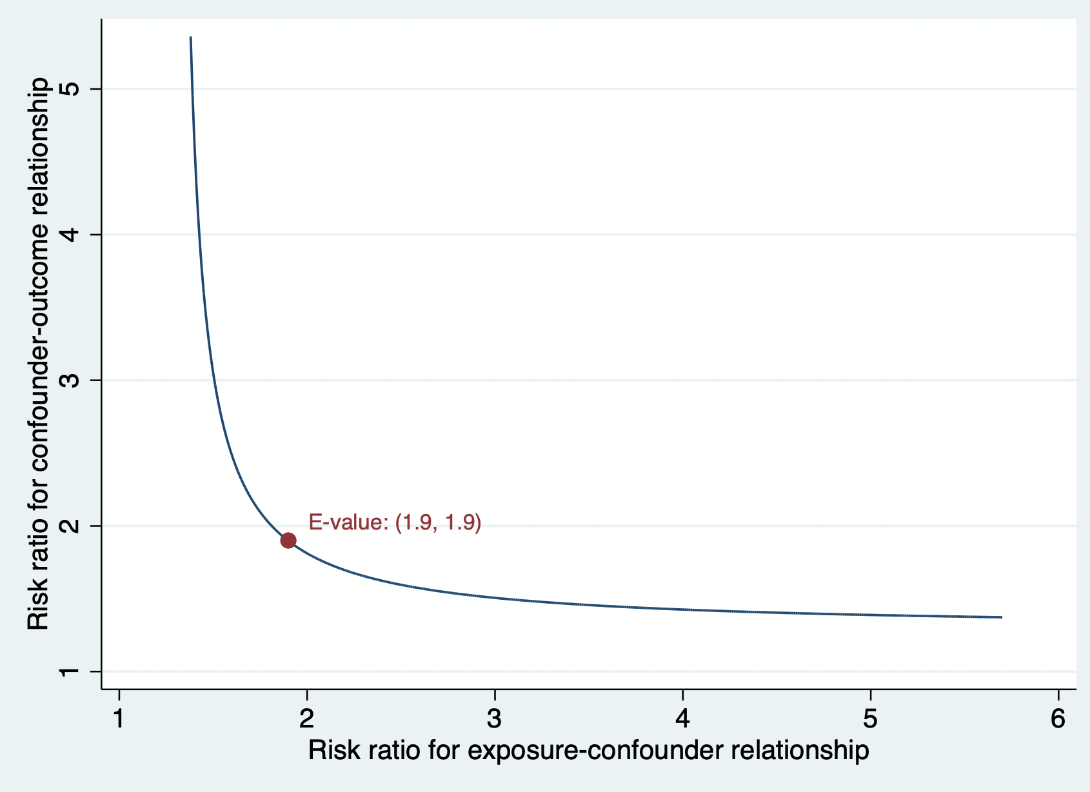


**Pooled point estimate of AEDC primary outcome (DV2) result:**

RR 0.97 (95% CI 0.77-1.25)

**Hypothetical estimate of minimum RR to indicate harmful casual effect of IVF conception:**

RR 1.26

E-values are useful to quantify the potential strength and prevalence of unmeasured bias required to change conclusions^1^. We utilised this measure to estimate the strength of unmeasured bias required to move the point estimate of relative risk toward an estimated significant harmful effect of RR equal to 1.26 is 1.9. The interpretation of this is as follows: a confounder that was associated with the exposure (IVF-conception) by less than 1.9-fold could result in the point estimate indicating a harmful effect of the exposure if it was associated with an increased risk of developmental vulnerability by more than 1.9-fold, but not less.

AEDC – Australian Early Development Census; DV2 – developmentally vulnerable in two or more AEDC domains

**Fig B – Sensitivity Analysis NAPLAN Primary Outcome – E-value Estimation**


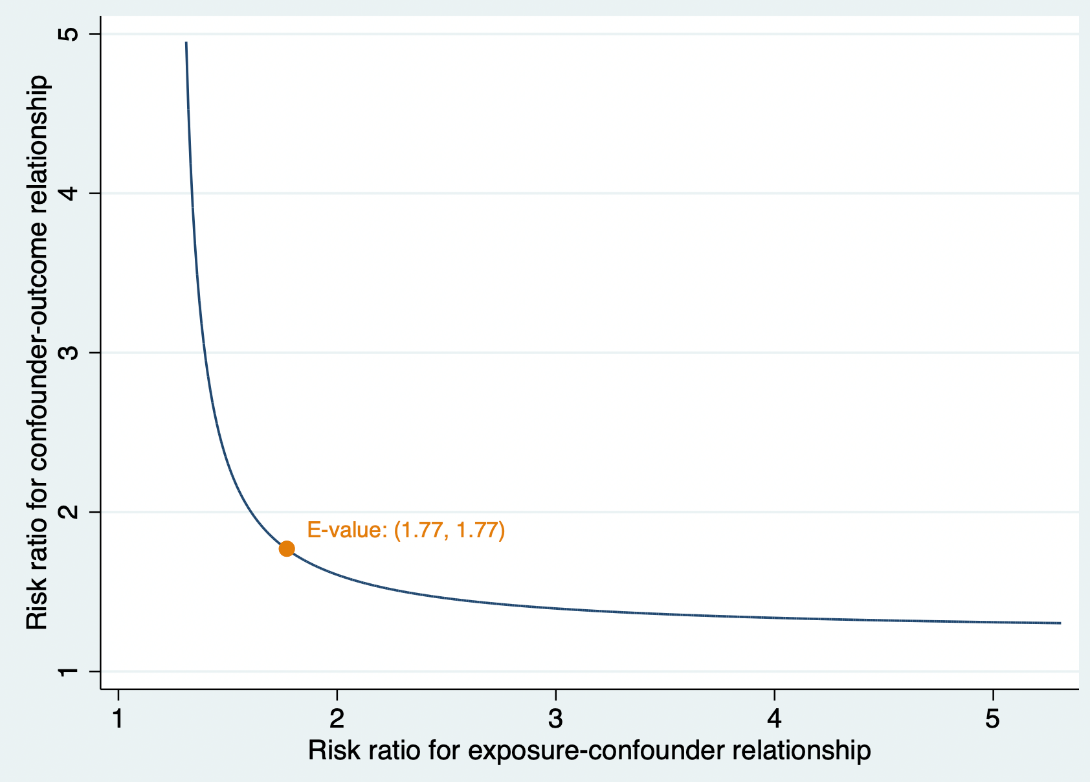


**Pooled point estimate of NAPLAN primary outcome (Overall Z-score) result:**

MD 0.030 (-0.018 to 0.077)

**Hypothetical estimate of minimum MD to indicate harmful casual effect of IVF conception:**

MD -0.20

An e-value was also calculated to estimate the strength of unmeasured bias required to move the point estimate of the overall NAPLAN z-score toward a clinically significant (lower) z-score for the IVF conceived, with a mean difference of greater than 0.2 standard deviations. The interpretation of this is as follows: a confounder that was associated with the exposure (IVF-conception) by less than 1.8-fold could result in the point estimate indicating a harmful effect of the exposure if it was associated with an increased risk of developmental vulnerability by more than 1.8-fold, but not less.

NAPLAN – National Assessment Program – Literacy and Numeracy

1. VanderWeele TJ, Ding P. Sensitivity Analysis in Observational Research: Introducing the E-Value. *Ann Intern Med* 2017;167(4):268-74. doi: 10.7326/M16-2607 [published Online First: 20170711]
