# Supplementary material for: School-age outcomes among IVF-conceived children: A population-wide cohort study
Source: PLoS Med. 2023 Jan 24;20(1):e1004148. doi: 10.1371/journal.pmed.1004148 (PMC9873192; doi:10.1371/journal.pmed.1004148)
Supplement: S12 File — Tables A and B. Table A. Traditional regression and treatment effect models (AEDC). Table B. Traditional regression and treatment effect models (NAPLAN). (DOCX) [file pmed.1004148.s013.docx]

**Table A: Traditional regression and treatment effect models (Australian Early Development Census, AEDC)**

| **Overall outcome**  **(Vulnerable on two or more domains)** | **Control** | **IVF** | **Regression co-efficient:** | |
| --- | --- | --- | --- | --- |
| **Raw data (n = 172,581)** | | | | |
|  | **Proportions** | | **Risk difference (95%CI, p-value)** | **Relative risk (95%CI, p-value)** |
| Standard logistic regression* | 0.140 | 0.104 | -0.036 (-0.45 to -0.027, p < 0.001)^c^ | 0.74 (0.68 to 0.81, p < 0.001)^c^ |
| **Covariate complete case - (n = 117,243), number of clusters = 101,826** | | | | |
|  | **Proportions** | | **Risk difference (95%CI, p-value)** | **Relative risk (95%CI, p-value)** |
| Standard logistic regression – Unadjusted* | 0.137 | 0.106 | -0.031 (-0.042 to -0.021, p < 0.001)^c^ | 0.74 (0.66 to 0.83, p < 0.001)^c^ |
| Standard logistic regression – Adjusted | 0.136 | 0.149 | 0.013 (-0.0015 to 0.026, p = 0.079) | 1.12 (0.99 to 1.26, p = 0.070) |
|  | **Predicted outcome proportions** | | **ATE Risk difference (95%CI, p-value)** | **ATE Relative risk (95%CI, p-value)** |
| Regression adjustment^ | 0.136 | 0.119 | -0.018 (-0.038 to 0.003, p = 0.095) | 0.87 (0.73 to 1.04, p = 0.119) |
| Doubly Robust IPWRA^^#^ | 0.137 | 0.127 | -0.010 (-0.040 to 0.020, p = 0.526) | 0.93 (0.73 to 1.18, p = 0.541) |
| IPW ^##^ | 0.137 | 0.113 | -0.023 (-0.047 to 0.000, p = 0.050) | 0.83 (0.67 to 1.02, p = 0.075) |
| AIPW ^^#^ – OLS | 0.137 | 0.121 | -0.016 (-0.041 to 0.009, p = 0.215) | 0.88 (0.72 to 1.09, p = 0.244) |
| AIPW ^^#^ – NLS | 0.137 | 0.120 | -0.017 (-0.042 to 0.008, p = 0.190) | 0.88 (0.71 to 1.08, p = 0.221) |
| **Multiple imputation (m=20) (n = 173,200) number of clusters = 156,747** | | | | |
|  | **Predicted outcome proportions** | | **ATE Risk difference (95%CI, p-value)** | **ATE Relative risk (95%CI, p-value)** |
| Regression adjustment ^ | 0.139 | 0.117 | -0.022 (-0.038 to -0.005, p = 0.013)^c^ | 0.84 (0.73 to 0.97, p = 0.022)^c^ |
| Doubly Robust IPWRA ^^#^ | 0.139 | 0.133 | -0.006 (-0.035 to 0.023, p = 0.697) | 0.95 (0.77 to 1.19, p = 0.702) |
| IPW ^#^ | 0.139 | 0.118 | -0.022 (-0.044 to 0.001, p = 0.066) | 0.84 (0.70 to 1.03, p = 0.091) |
| AIPW ^^#^ – OLS | 0.139 | 0.124 | -0.015 (-0.039 to 0.009, p = 0.216) | 0.89 (0.74 to 1.08, p = 0.242) |
| AIPW ^^#^ – NLS | 0.139 | 0.124 | -0.015 (-0.040 to 0.009, p = 0.209) | 0.89 (0.73 to 1.08, p = 0.236) |
| NN Match |  |  | -0.021 (-0.037 to -0.005, p = 0.012)^c^ |  |
| PS Match^#^ |  |  | -0.015 (-0.045 to 0.017, p = 0.368) |  |

*Complete case differs from raw data as complete limited to outcome mean in observations with no missing covariates. ^ outcome models: sex at birth, age at assessment, language background other than English, socioeconomic status, maternal age, parity and education, second parent education. # Treatment assignment models: sex at birth, age at assessment, language background other than English, socioeconomic status, maternal age, parity and education.

c: 95% confidence interval does not cross the null

SE: standard error, IPW: inverse probability weight, AIPW: augmented IPW, OLS: Ordinary least squares, NLS: Non-linear least squares, NN Match: nearest neighbour match, PS Match: propensity score match.

**Table B: Traditional regression and treatment effect models (National Assessment Program – Literacy and Numeracy, NAPLAN)**

| **Overall Outcome**  **(Overall NAPLAN score z-score)** | **Control** | **IVF** | **Regression co-efficient:** | **(95% CI, p value)** |
| --- | --- | --- | --- | --- |
| **Raw data (n = 316,377)** | | | | |
|  | **Means (SE)** | | **Mean difference (SE)** |  |
| Standard linear regression* | -0.006 (0.002) | 0.232 (0.010) | 0.238 (0.011) | (0.217 to 0.259, p < 0.001)^c^ |
| **Covariate complete case (n = 263,335), number of clusters = 200,777** | | | | |
|  | **Means (SE)** | | **Mean difference (SE)** |  |
| Standard linear regression – unadjusted* | 0.047 (0.002) | 0.243 (0.011) | 0.195 (0.012) | (0.172 to 0.218, p < 0.001)^c^ |
| Standard linear regression – adjusted | 0.053 (0.002) | 0.029 (0.011) | -0.023 (0.011) | (-0.045 to -0.003, p = 0.028)^c^ |
|  | **Predicted outcome means (SE)** | | **ATE mean difference (SE)** |  |
| Regression adjustment^ | 0.053 (0.002) | 0.080 (0.019) | 0.027 (0.019) | (-0.010 to 0.064, p = 0.147) |
| Doubly robust IPWRA^^#^ | 0.052 (0.002) | 0.038 (0.028) | -0.014 (0.028) | (-0.070 to 0.041, p = 0.610) |
| IPW ^#^ | 0.052 (0.002) | 0.023 (0.039) | -0.029 (0.039) | (-0.106 to 0.047, p = 0.452) |
| AIPW ^^#^ – OLS | 0.052 (0.002) | 0.055 (0.033) | 0.003 (0.032) | (-0.061 to 0.068, p = 0.921) |
| AIPW ^^#^ – NLS | 0.052 (0.002) | 0.055 (0.033) | 0.003 (0.032) | (-0.061 to 0.068, p = 0.921) |
| **Multiple imputation (m=20) ^^ (n = 342,311), number of clusters = 255,858** | | | | |
|  | **Predicted outcome means (SE)** | | **ATE mean difference (SE)** |  |
| Regression adjustment^^^ | -0.015 (0.002) | 0.032 (0.019) | 0.047 (0.018) | (0.012 to 0.083, p = 0.009)^c^ |
| Doubly robust IPWRA^^#^ | -0.016 (0.002) | 0.005 (0.028) | 0.011 (0.028) | (-0.044 to 0.066, p = 0.687) |
| IPW ^##^ | -0.016 (0.002) | -0.022 (0.035) | -0.006 (0.035) | (-0.075 to 0.063, p = 0.867) |
| AIPW ^^#^ – OLS | -0.016 (0.002) | 0.008 (0.031) | 0.024(0.031) | (-0.036 to 0.084, p = 0.044) |
| AIPW ^^#^ – NLS | -0.016 (0.002) | 0.008 (0.031) | 0.024(0.031) | (-0.036 to 0.084, p = 0.044) |
| Nnmatch |  |  | 0.001 (0.014) | (-0.027 to 0.030, p = 0.928) |
| PSmatch |  |  | 0.003 (0.032) | (-0.060 to 0.065, p =0.937) |

*Complete case differs from raw data as complete limited to outcome mean in observations with no missing covariates.

^ outcome models: sex at birth, age at assessment, language background other than English, socioeconomic status, maternal age, parity and education, second parent education. #Treatment assignment models: sex at birth, age at assessment, language background other than English, socioeconomic status, maternal age, parity and education, second parent education c: 95% confidence interval does not cross the null

Abbreviations: SE: standard error, IPW: inverse probability weight, AIPW: augmented IPW, OLS: Ordinary least squares, NLS: non-linear least squares NN Match: nearest neighbour match, PS Match: propensity score match.
