# Supplementary material for: School-age outcomes among IVF-conceived children: A population-wide cohort study
Source: PLoS Med. 2023 Jan 24;20(1):e1004148. doi: 10.1371/journal.pmed.1004148 (PMC9873192; doi:10.1371/journal.pmed.1004148)
Supplement: Attachment — Submitted filename: PLOSMed_Response_IVF_2022_FINAL.docx [file pmed.1004148.s015.docx]

Response regarding "School-age outcomes among IVF-conceived children: a causal inference analysis using linked population-wide data" (PMEDICINE-D-22-02257R1).

Dear Editors, *PLOS Medicine*,

Thank you for the opportunity to respond the reviewer’s comments. Here, we provide responses to all the Editors and reviewers’ comments. Where relevant, we show the new additions to the manuscript in this rebuttal, with changes highlighted in green.

We have also uploaded a revised version of our manuscript with changes also highlighted in green.

Thank you.

Kind regards,

Dr Anthea Lindquist, corresponding author.

**Editors Comments:**

**ABSTRACT**
Abstract Methods and Findings:

1) Please quantify the main results with 95% CIs and p values.

*As requested, we have now included 95% confidence intervals and p-values in the abstract.*

2) Please include the important dependent variables that are adjusted for in the analyses.

*Done. An additional sentence has been added to the Abstract Methods, as below (line 59-60):*

The models were adjusted for sex at birth, age at assessment, language background other than English, socioeconomic status, maternal age, parity and education.

3) In the last sentence of the Abstract Methods and Findings section, please describe the main limitation(s) of the study's methodology.

*Done. We now include the following (line 61-63):*

Study limitations included the use of observational data, the potential for unmeasured confounding, the presence of missing data and the necessary restriction of the cohort to children attending school.

At this stage, we ask that you include a short, non-technical Author Summary of your research to make findings accessible to a wide audience that includes both scientists and non-scientists. The Author Summary should immediately follow the Abstract in your revised manuscript. This text is subject to editorial change and should be distinct from the scientific abstract. Please see our author guidelines for more information: <https://journals.plos.org/plosmedicine/s/revising-your-manuscript#loc-author-summary>

*4) We have now included the following summary, as per PLOS Medicine guidelines:*

**PLAIN LANGUAGE SUMMARY**

**Why was this study done?**

- More than 8 million children have been conceived globally with the assistance of in-vitro fertilisation (IVF).
- Some studies suggest these children have an increased risk of birth defects, congenital abnormalities, autism spectrum disorder, developmental-delay and intellectual disability.
- Educational and school aged developmental outcomes following IVF conception have not yet been adequately characterised.

**What did the researchers do and find?**

- Using state-wide, linked population data from Victoria, Australia, we investigated the school-aged developmental and educational outcomes for children born following IVF assisted conception.
- The study examined two separate assessments of school aged development and educational outcomes among 585,659 children, including 11,059 children who were conceived via IVF.
- This study was designed and performed within a causal framework, in order to produce the best possible estimate of exposure effect using observational data.
- We found no difference in school aged childhood developmental and educational outcomes between IVF and spontaneously conceived children.

**What do these findings mean?**

- These findings provide reassurance for current and prospective parents, and clinicians alike who are involved in IVF.
- This information may be useful in providing informed consent and education to those considering IVF, and those with children conceived via IVF.

**INTRODUCTION**
5) Please conclude the Introduction with a clear description of the study question or hypothesis.

*We have now included our study hypothesis at the end of the Introduction (line 137-140), as below:*

Our study hypothesised that using a causal inference approach and employing the necessary assumptions, there is no causal effect of IVF conception on childhood developmental or educational outcomes.

**METHODS and RESULTS**

6) Please provide 95% CIs and p values where relevant. When a p value is given, please specify the statistical test used to determine it.

*95% confidence intervals and p values have now been added throughout the text (highlighted in green) and to Tables 2 to 4). As requested, the tests used to determine the p values have now been added to the text and table legends.*

**GENERAL**
7) In the context of the reviewer comments below, it might be necessary to revise your title
Please do so according to PLOS Medicine's style. Your title must be non-declarative and not a question. It should begin with main concept if possible.

"Effect of" should be used only if causality can be inferred, i.e., for an RCT. Please place the study design ("A randomized controlled trial," "A retrospective study," "A modelling study," etc.) in the subtitle (ie, after a colon)

*As requested, our title is non-declarative. We do not use the words ‘effect of’. Regarding the general issue of causal language, please see our first response to academic reviewer 1.*

8) Please ensure that the study is reported according to the STROBE guideline and include the completed STROBE checklist as Supporting Information. Please add the following statement, or similar, to the Methods: "This study is reported as per the Strengthening the Reporting of Observational Studies in Epidemiology (STROBE) guideline (S1 Checklist)." The STROBE guideline can be found here: <http://www.equator-network.org/reporting-guidelines/strobe/> When completing the checklist, please use section and paragraph numbers, rather than page numbers.

*Thank you, a completed STROBE guideline has now been included with the updated documents we’ve uploaded.*

9) Thank you for including a prospective analysis plan, please include any changes made to the analyses- including those made in response to peer review comments- in the Methods section of the paper, with rationale.

*No further changes have been made to our pre-specified analyses based on peer review comments.*

10) In the manuscript text, please indicate: (1) the specific hypotheses you intended to test, (2) the analytical methods by which you planned to test them, (3) the analyses you actually performed, and (4) when reported analyses differ from those that were planned, transparent explanations for differences that affect the reliability of the study's results. If a reported analysis was performed based on an interesting but unanticipated pattern in the data, please be clear that the analysis was data-driven.

*We have now included a clear hypothesis in our Introduction, as stated above (line 137-142). Our causal methods are now much more clearly outlined in Methods, with an additional explanation about the additional steps taken following initial analysis (line 239-264), as below:*

Analysis involved detailed examination of missing data, consideration of missing outcome data, multiple imputation of missing covariate data, consideration of clustering within mothers and adjustments to effect size modelling (S5 Methods). All covariates in the analysis model that had missing data were imputed, even if missing was very low. The proportions of missing data are described in Table 1. For the AEDC analysis, imputed covariates included parity, age at assessment, maternal education, SEIFA and outcome score. For the NAPLAN analysis, imputed covariates included parity, age at assessment, maternal education, paternal education, SEIFA and outcome score. Only paternal education for AEDC, and BMI for both AEDC and NAPLAN, were excluded from imputation and analysis because the missingness was too high (> 40%).

In our study, diagnostic tests performed after planned treatment effect modelling (Supplementary file 9 – IPW Overlap) demonstrated poor overlap of exposed and non-exposed cohorts. We therefore restricted our analysis to the IVF population whose weights overlapped with the control group (S5 Methods).

A doubly robust inverse-probability-weighted regression adjustment (IPWRA) model^44,45^ was then used to estimate a) the risk difference (RD) and relative risk (RR) for binary outcomes (AEDC and NAPLAN) and b) mean differences (MD) for continuous outcomes (NAPLAN z-score). Clustering of data within mothers due to more than one singleton birth during the study period was accounted for in the imputation models, the calculation of inverse probability weights and estimation of the treatment effect by using robust standard errors. Provided the assumptions outlined above are satisfied, the estimates generated from these analyses can be interpreted as the population average causal effect, that is, the mean effect on the outcome if the treatment was applied to the entire population and contrasted with the outcome if the entire population received the control condition.

DISCUSSION
11) Please remove sub-headings such that the discussion is presented follows: a short, clear summary of the article's findings; what the study adds to existing research and where and why the results may differ from previous research; strengths and limitations of the study; implications and next steps for research, clinical practice, and/or public policy; one-paragraph conclusion.

*All sub-headings have now been removed.*

**Comments from the Academic Editor#1:**

1) I do not buy the causal language. All observational studies have potential for unmeasured confounders. I think the use and emphasis of causal language could actually distract attention from the main message, which I think is important. In fact, the unadjusted data indicate an apparent protective effect of IVF which disappears on adjustment (Table 2). It would be good to add 95% CI to the unadjusted analysis to see if this was beyond the play of chance. But it is apparent that measured parental characteristics are associated with an increased chance of using IVF and a decreased risk of poor educational outcome. Hence, it follows that unmeasured parental characteristics associated with an increased chance of using IVF and a decreased risk of poor educational outcome could be masking an adverse effect of IVF and the use of causal language is wrong.

*Here, we propose responding to the general issue of causality (and consequently, the language used to describe our work) by drawing in the views of all reviewers.*

*Unlike most studies in the field, we approached our observational dataset from a causal analysis perspective, and we embarked on rigorous methodology that complied with all necessary requirements of this approach. This included preparing a pre-specified statistical analysis plan and a comprehensive Directed Acyclic Graph, devising a target trial emulation (S2 Table 1), employing the appropriate causal assumptions, handling of missing data, inverse probability weighting of all covariates and trimming of our cohorts to ensure overlap of the populations being compared, and then estimating the effect of treatment on the outcome. We appreciate that these methods may not have been adequately stated in our initial manuscript and have now included additional detail in our Methods (line 214-233), including a new section on causal assumptions, in response to feedback from the Reviewers.*

*We wish to note that there seems to be generally strong support for our causal approach from the remaining reviewers. Importantly, the analysis we did using the casual framework seems to have the general approval of the expert statistical reviewer. The second academic editor has examined our methods and was satisfied that we have completed the appropriate procedures for causal inference to be made. Reviewer 3 said that our approach is new and should be applied more broadly to observational data.*

*However, in light of the concerns raised by the first academic editor (and also following some suggestions from the second academic editor), we have significantly toned down the language of causation. In this revision, we are more circumspect in the description of our study findings throughout the manuscript (in claiming causation).*

*As requested, we have added 95%CI to our text and tables and have outlined much more clearly our analytical methods, accounting for potential confounding, and the causal assumptions we employed.*

2) It would be good to have a positive control, i.e. data on a factor which we know is associated with educational outcome. That way we know their methods can detect an association if there is one there. Preterm birth is an obvious one. Perhaps an easy way to do this is to provide coefficients for all the covariables that they included in adjustment.

*Our response to this point is combined with our response to point 3. Please see below.*

3. It would actually be quite interesting to see what the results are if they applied a more commonly used method of multivariate adjustment, such as logistic regression or propensity score analysis just to see how different (if at all) the results are with the method they employ. Comments 2 and 3 could be addressed by providing a supplementary table of unadjusted and adjusted ORs from logistic regression to allow comparison.

*Thank you for this suggestion. As requested by the reviewer, we have now included two tables (S16 Tables 9 and 10) in our supplementary files that detail estimated treatment effects for each analysis model. We present results for the following 7 models: unadjusted analyses using raw and complete case data, adjusted models including multivariable regression, regression adjustment (G-computation), IPW treatment assignment model, AIPW and doubly-robust IPW regression adjustment using multiply imputed data.*

*In response to the reviewers’ comments, it is noteworthy as shown in supplementary Tables 9 and 10 that the unadjusted logistic regression analyses of raw and complete case data suggested a protective effect of the exposure; however, both models are known to produce biased estimates when the missingness mechanism is not Missing Completely at Random (see excerpt from Stuart et al 2010 below).*

*"A number of authors, including Cochran and Rubin (1973); Rubin* *(1973a,b, 1979); Heckman et al. (1998b); Rubin and Thomas (2000), and Rubin (2001), have* *shown that methods such as linear regression adjustment can actually increase bias in the* *estimated treatment effect when the true relationship between the covariate and outcome is* *even moderately non-linear, especially when there are large differences in the means and* *variances of the covariates in the treated and control groups".*

4. I agree with the comments of the statistician. But it is reassuring that their overall assessment is positive.

*Thank you. We appreciate the positive statistical review.*

**Comments from Academic Editor#2**

1) I like this one, and find the attempt to use a causal inference framework interesting. I think the methodology is well described, including the emulation trial details and DAG (I agree with reviewer 3 that these may benefit from being in main paper rather than supplementary to help clarity). I think that Gestational age at birth, mode of delivery and birthweight should be shown in the DAG as potential mediators for clarity.

*Thank you for the positive feedback. We have included a reference to the target trial emulation table in the main text of the manuscript (as below) and agree that it is important to the understanding of our study.*

To ensure the assumptions underlying our causal approach were as robust as possible, we considered our observational data in direct comparison with the conditions of a target trial (S2 Table 1).

*The reviewer (and reviewer 3) suggest we add emulation trial details and the DAG to the main manuscript. However, the trial emulation table is over 4-pages long. We feel it would be overly cumbersome to include it in the main manuscript. We are happy to include it if the editor requests we do so.*

*Our DAG was pre-specified and we are therefore reluctant to alter it post-hoc. However, we have clarified in the Methods that gestational age, mode of birth and birthweight were not included in the model, and why (line 194-196, as below).*

Gestational age at birth, mode of delivery and birthweight were considered to be mediators on the causal pathways of interest and therefore not adjusted for in this analysis.

2) I think the conclusions could be more circumspect- stating that in this analysis (given the assumptions made) we found no evidence of a causal association…Also would be useful to highlight the need for replication in other cohorts to strengthen the evidence base.

*Thank you for this feedback. As requested, we have adjusted our language throughout the manuscript so it is indeed more circumspect. We have also added a line in the discussion that highlights the need for others to replicate our findings (line 430-431), as below:*

It is important that our methods are replicated in future studies to strengthen the existing evidence base.

3) It would also be good if the authors could discuss generalisability as a limitation, given the large drop off in numbers included in the analyses.

*We recognise the reduction in numbers resulting from our trimming of datasets. However, this step was necessary to ensure overlap of our cohorts. We have now included a paragraph in the limitations section of our Discussion identifying this issue (466-471), as below:*

Generalisation of our findings to all IVF births is a potential study limitation. As described in our Methods, observations with non-overlapping propensity scores were excluded from analysis in order to meet the assumption of positivity, required for causal inference under the potential outcomes framework. Generalisation of our findings to all IVF births therefore requires the consideration that the baseline characteristics of the population of interest are comparable to the IVF cases analysed in our final cohort.

4) I quite like the causal inference approach. Sure, there may be unmeasured confounding but that doesn’t invalidate the methods used and the authors are clear about assumptions made, and discuss unmeasured confounding.

*Thank you for the positive feedback.*

5) A positive control may be useful – but would need some careful thought. I am not sure that preterm birth fits with the emulation trial methodology (you cannot ‘randomise’ to preterm birth…). Gestation at birth is on the causal pathway between IVF and the educational outcome so it was not included as a covariate as it would cause collider bias- this seems appropriate. Including all coefficients as stated would lead to “Table 2 Fallacy.

*Thank you for this comment. We agree that preterm birth and gestational age at birth are post-exposure covariates in this analysis and are also on the causal pathway, and cannot therefore be included in the adjusted model. As suggested by the first academic editor, we have now included a table in our supplementary files that details all potential unadjusted and adjusted model estimates.*

6) Reasonable to do a sensitivity analysis with a different methodology (but including same adjustment covariates). This may increase confidence in the findings. Note this would be post hoc.

*We already conducted and included the sensitivity analyses we felt were pertinent to our findings (Supplementary Tables 4-8 and Figures 13 and 14). Of note, they were all pre-specified: we did not pursue any post-hoc analyses, outside those pre-specified in our SAP.*

**Comments from the reviewers:**

**Reviewer #1 (methodological reviewer):** This aim of this study was to determine the causal effect of in-vitro fertilization (IVF) conception on primary school-age childhood developmental and educational outcomes, compared with outcomes following spontaneous conception.

1) "Covariates to be considered for inclusion in the statistical analysis models were decided a priori by the authorship team whose expertise included epidemiology, perinatology, reproductive endocrinology and education". The authors have satisfactorily adjusted for potential confounding in the analysis.

*Thank you for the positive comment.*

2) "For the pre-specified statistical analysis plan (SAP) and the directed acyclic graph (DAG), see supplementary file (eFigure 1 in S3)." The authors have suitably provided the DAG in the supplementary material. Can the authors also please provide a copy of the SAP in the supplementary material (it is not currently clear if or where this is attached to the file)?

*Thank you. We apologise for this oversight and have now included the SAP in the supplementary files.*

3) "A doubly robust inverse-probability-weighted regression adjustment (IPWRA) model43,44 was used...". The authors have applied technically appropriate and rigorous statistical methods within the context of this research.

*Thank you. This approach was guided by review of the current literature and rigorous input from our Biostatistician co-authors (RJH and LG).*

4) "Provided assumptions are satisfied, the estimates generated from these routines can be interpreted as the population average causal effect". Can the authors please include a transparent and thorough discussion on the assumptions that are required for causal inferences to be drawn?

*Assumptions were carefully considered throughout the course of planning our analysis. As requested, we have now included a comprehensive summary of causal assumptions in a new section of our Methods (line 214-233). We agree this enhances the readability of our manuscript and thank the reviewer for this suggestion.*

**Causal assumptions**

The ATE (Average Treatment Effect) estimand used in this study is based upon the Potential Outcomes Framework. If a set of assumptions is met, then causal interpretation can be made. The three assumptions include counterfactual consistency, ignorability (conditional exchangeability) and positivity. Counterfactual consistency means that the definition of exposure is consistent for all individuals. Ignorability states that treatment assignment can be considered random after controlling for, conditioning on, a set of covariates^43^. By identifying confounding variables, and importantly, the structure of the relationships between variables, via a Directed Acyclic Graph (S3 Fig 1) and by performing appropriate statistical modelling to balance the population (for example, inverse probability weighting), observed populations can be considered exchangeable or “unconfounded”. Exchangeability requires that there are no important unmeasured confounders; this assertion is untestable. The positivity assumption means that for all observations, the conditional probability of being exposed (receiving treatment/no treatment) is greater than zero. This is likely violated if overlap of the control and exposed populations is poor^26^.

To best emulate a target trial, it must be possible for all participants to potentially receive both treatments. To ensure the assumptions underlying our causal approach were as robust as possible, we considered our observational data in direct comparison with the conditions of a target trial (S2 Table 1).

*We have also added the following phrase to the Discussion and Conclusion, “under the specified assumptions”, to ensure our findings are interpreted within the context of the analysis performed.*

5) "Analysis involved detailed examination of missing data, consideration of missing outcome data, multiple imputation of missing covariate data, consideration of clustering within mothers and adjustments to effect size modelling.  Finally, sensitivity analysis was also performed to address identified sources of potential bias." Can the authors please provide more detail on these important analyses within the main article here?

*Thank you for this comment. As requested, we have added further detail to the relevant sections in Methods (the additions are shown below). These additions now clarify which missing covariate data were imputed for each model, our management of clustering and effect size modelling. Our sensitivity analyses are extensive and have been outlined in detail in Supplementary files.*

**Statistical Analysis**

Descriptive statistics were calculated and are reported for each cohort by IVF exposure status, according to type and distribution of data.

Analysis involved detailed examination of missing data, consideration of missing outcome data, multiple imputation of missing covariate data, consideration of clustering within mothers and adjustments to effect size modelling (S5 Methods). All covariates in the analysis model that had missing data were imputed, even if missing was very low. The proportions of missing data are described in Table 1. For the AEDC analysis, imputed covariates included parity, age at assessment, maternal education, SEIFA and outcome score. For the NAPLAN analysis, imputed covariates included parity, age at assessment, maternal education, paternal education, SEIFA and outcome score. Only paternal education for AEDC and BMI for both AEDC and NAPLAN were excluded from imputation and analysis because the missingness was too high (> 40%).

In our study, diagnostic tests performed after planned treatment effect modelling (S9 Fig 6-9) demonstrated poor overlap of exposed and non-exposed cohorts. We therefore restricted our analysis to the IVF population whose weights overlapped with the control group (S5 Methods).

A doubly robust inverse-probability-weighted regression adjustment (IPWRA) model^44,45^ was then used to estimate a) the risk difference (RD) and relative risk (RR) for binary outcomes (AEDC and NAPLAN) and b) mean differences (MD) for continuous outcomes (NAPLAN z-score). Clustering of data within mothers due to more than one singleton birth during the study period was accounted for in the imputation models, the calculation of inverse probability weights and estimation of the treatment effect by using robust standard errors. Provided the assumptions outlined above are satisfied, the estimates generated from these analyses can be interpreted as the population average causal effect, that is, the mean effect on the outcome if the treatment was applied to the entire population and contrasted with the outcome if the entire population received the control condition.

Finally, sensitivity analyses were also performed to address identified sources of potential bias. These methods are detailed in the supplementary files (S5 Methods and S6 Fig 2).

6) "Overall, a total of 11,059 IVF-conceived children and 401,654 spontaneously conceived children were included in the study (2,614 IVF cases and 100,184 controls were in both study arms)."

Whilst the authors have examined that "Analysis of the linked and nonlinked cases showed little evidence of association between linkage and exposure status (p = 0.80); that is, IVF cases were just as likely to be included in the final linked cohort as controls", can the authors please further comment on whether the final included samples analysed in this study can be considered to be representative of the wider populations of interest within the context of this research?

*We estimate that our IVF database includes >95% of IVF cycles in the state of Victoria during the study timeframe, given we were able to access data from the three main private IVF providers in the state. Our control cohort included all births in the state during the study timeframe with >95% ascertainment for the years relating to the specific outcome metrics. We have now included these data in additional tables (S4 Tables 2A and 2B), which have been referenced accordingly in the text (line 281-282), as below. As such, we believe our findings are broadly generalisable.*

We estimate that our study cohort includes >95% of IVF conceptions during the study timeframe (S4 Tables 2A and 2B).

7) "Outcome data were missing for 5.6% of the AEDC-linked cohort. The vast majority (92%) of these missing cases were children with special needs (5.2% of overall cohort). There was no evidence of an association between the presence of missing outcome and exposure status (p = 0.68). Sensitivity analysis was performed by 1) excluding children with special needs and 2) including these children, with multiple imputation of their missing outcomes (see supplementary file: eTable 4 and 5 - Sensitivity Analysis (AEDC) in S11 and s12). "

and
"Spontaneously conceived children were more likely to have missing NAPLAN data (7.6%) than IVF-conceived children (5.9%, p<0.001). During the primary analysis, missing outcomes related to a child being absent or withdrawing from the test, were imputed. The results presented include 7,222 children who were exempt from sitting the NAPLAN, with their results set to the lowest possible outcome score. Sensitivity analysis was performed by 1) excluding these children and 2) including the exempt cases, with multiple imputation of their missing outcomes. There was no meaningful difference in the results (see supplementary file: eTable 6 and 7 - Sensitivity Analysis (NAPLAN) in S13 and S14). "

The authors have appropriately communicated and handled missing outcome data within the analysis.

*Thank you for the positive feedback.*

8) "Maternal education level was missing for 30.5% and maternal post-school education was missing for 31.6%."and "Second parent school education level was missing in 13.8% of cases and post-school education missing in 15.4% of cases. "

Whilst the authors discuss missing data in the discussion of the study limitations, can the authors please clarify and expand on how they dealt with missing covariate data in the Methods and Results sections?

*Thank you, we can now see we did not clearly describe which covariates and what range of missing data were handled using multiple imputation. We have now added these details to the Methods (line 241-248), as below:*

All covariates in the analysis model that had missing data were imputed, even if missing was very low. The proportions of missing data are described in Table 1. For the AEDC analysis, imputed covariates included parity, age at assessment, maternal education, SEIFA and outcome score. For the NAPLAN analysis, imputed covariates included parity, age at assessment, maternal education, paternal education, SEIFA and outcome score. Only paternal education for AEDC and BMI for both AEDC and NAPLAN were excluded from imputation and analysis because the missingness was too high (> 40%).

9) Overall, the results are presented clearly and the main study limitations have been thoroughly addressed.

*Thank you, we are grateful for this positive comment.*

**Reviewer #2:** The authors report the lack of an association of IVF with later childhood educational outcomes using routinely collected data from Australia. This is an extremely well conducted and well written study, with robust and appropriate sensitivity analyses which adds substantially to the field and will reassures many parents.

*Thank you for this positive feedback.*

Minor comments:

1) The authors make a big deal of the causal analysis, and I would suggest statistical review to confirm the appropriateness of the methodology and causal claims. I am conscious that the same used to be said regarding matching and that has now shown to be incorrect. I was not convinced that the paper would materially suffer by removing the "casual analysis" claims in the title or introduction etc.

*Thank you, we have taken this comment into consideration. The statistician has reviewed our methodology and provided positive feedback regarding our approach. We have therefore kept the causal language as is, but as suggested by the second academic editor, we been more circumspect in our language. Please see our comments to the Editor above.*

2) I wondered if graphical representation of the results would further enhance the paper.

*We thank the reviewer for this suggestion. We feel our manuscript already has many tables and supplementary files and we are reluctant to add more (we can do an overview pictorial if the editors request this). Additionally, we do not feel our estimation of a causal effect lends itself well to graphical representation.*

**Reviewer #3:** This study examined associations of ART conception with School-age outcomes using register data from Australia. The authors should be commended for trying to provide more reliable causal evidence from observational data, something we should all be doing.

*Thank you for this positive feedback, we agree that this approach should be used more in the analyses of observational data, where the necessary assumptions can be met.*

I have only a few minor comments.

1) eTable 1 should be brought into the main paper and / or a separate section in the methods should be added on the target trial emulation procedure.

*This point was addressed in our response to the second academic editor. Running over a few pages, we feel the table itself is too large to add to the main table (but can do so if the editor requests). Instead, we have now referenced the table in our manuscript (line 230-233), as below:*

To best emulate a target trial, it must be possible for all participants to potentially receive both treatments. To ensure the assumptions underlying our causal approach were as robust as possible, we considered our observational data in direct comparison with the conditions of a target trial (S2 Table 1).

2) Where is the study protocol published / date it was published?

*There was no study protocol published for this study. However, the pre-specified SAP was agreed upon by all contributing authors and signed off by all in May 2020, prior to the commencement of data analysis. This is now clearly outlined in Methods (line 196-198, as below) and the SAP is now included in the supplementary files.*

Our pre-specified statistical analysis plan (SAP) and the directed acyclic graph (DAG) were agreed upon and signed off by all authors in May 2020, prior to the commencement of data analysis (S3 Protocol and S3 Fig 1).

3) In line with the glossary, the exposure of interest should be referred to as ART rather than IVF.

*Our study exposure did not include all techniques within the overarching label of assisted reproductive technology (ART), such as ovulation induction, intra-uterine insemination (IUI) and In-Vitro Fertilisation. Our prespecified exposure of interest included only children born following IVF conception, as detailed in our Introduction and Methods. We have therefore elected to keep our exposure as IVF as we believe this is the most accurate.*

4) The aim of the study was to emulate a target trial however, baseline characteristics differed considerably between the treatment groups. Can you describe the implications of this. Does it mean that trial emulation failed? Is there a way you can try to fix this?

*The exposed and non-exposed groups indeed differed in their baseline characteristics – this was expected. Our statistical methods aimed to emulate a target trial by ‘balancing out’ these baseline differences. The result is that it minimises the chance of bias arising from differences in baseline characteristics in our overall estimate of risk. Hence, trial emulation did not ‘fail’ in any way – the statistics were done successfully, and to high fidelity.*

*These methods have now been clarified in a separate section of the Methods (line 214-233) where we outline the causal assumptions, including overlap and positivity, underpinning our analysis, as below:*

**Causal assumptions**

The ATE (Average Treatment Effect) estimand used in this study is based upon the Potential Outcomes Framework. If a set of assumptions is met, then causal interpretation can be made. The three assumptions include counterfactual consistency, ignorability (conditional exchangeability) and positivity. Counterfactual consistency means that the definition of exposure is consistent for all individuals. Ignorability states that treatment assignment can be considered random after controlling for, conditioning on, a set of covariates^43^. By identifying confounding variables, and importantly, the structure of the relationships between variables, via a Directed Acyclic Graph (Supplementary eFigure 1) and by performing appropriate statistical modelling to balance the population (for example, inverse probability weighting), observed populations can be considered exchangeable or “unconfounded”. Exchangeability requires that there are no important unmeasured confounders; this assertion is untestable. The positivity assumption means that for all observations, the conditional probability of being exposed (receiving treatment/no treatment) is greater than zero. This is likely violated if overlap of the control and exposed populations is poor^26^.

To best emulate a target trial, it must be possible for all participants to potentially receive both treatments. To ensure the assumptions underlying our causal approach were as robust as possible, we considered our observational data in direct comparison with the conditions of a target trial (Supplementary eTable 1).

5) If data are available, it would be of interest to present results separately for fresh and frozen embryo transfers. Please consider this.

*We thank the reviewer for this insightful suggestion. Such a comparison was beyond the scope of the current study. To emulate a target trial, we needed to select two populations to compare – we opted to focus on IVF vs non-IVF conceived children.*

*We agree that it would be interesting to now compare childhood outcomes after fresh and frozen embryo transfer. Planning is already underway for such a subsequent analysis. We plan to run another target emulation trial, which requires a full pre-specified SAP etc. (had we done this as a simple analysis in this first paper, the findings could only be considered exploratory or ‘hypothesis generating’).*
