# Supplementary material for: School-age outcomes among IVF-conceived children: A population-wide cohort study
Source: PLoS Med. 2023 Jan 24;20(1):e1004148. doi: 10.1371/journal.pmed.1004148 (PMC9873192; doi:10.1371/journal.pmed.1004148)
Supplement: Attachment — Submitted filename: PlosMED_Revisions2.0.docx [file pmed.1004148.s016.docx]

**Response regarding "School-age outcomes among IVF-conceived children: a causal inference analysis using linked population-wide data" (PMEDICINE-D-22-02257R1).**

Dear Editors, PLOS Medicine,

Thank you very much for the further feedback and opportunity to provide an updated version of our manuscript. We have responded the Editors and reviewers’ comments below. The original revisions remain highlighted in the manuscript in green, and the new changes are highlighted in yellow. The relevant excerpts have also been included here.

Thank you.

Kind regards,

Dr Anthea Lindquist (corresponding author)

**Requests from Editors:**
GENERAL
Please address all editor and reviewer comments detailed below, in full.

1) Please remove the funding/financial disclosure

*The funding and financial disclosure statement has now been removed. Relevant funding disclosures are contained within the online submission form.*

DATA AVAILABILITY STATEMENT
2) Please provide a URL for The Centre for Victorian Data Linkage

*The data availability statement has been updated (lines 607-611) and now reads:*

Data for this study was provided by various data custodians and linked by the Centre for Victorian Data linkage (https://www.health.vic.gov.au/reporting-planning-data/the-centre-for-victorian-data-linkage). With relevant ethical approval, data are available upon request to the governing data custodians.

AUTHOR SUMMARY
3) Thank you for including an author summary. 
Line 74: Consider an alternative term to “defect” perhaps “congenital anomalies” in place of “…birth defects, congenital abnormalities…” to account for both structural and functional anomalies, or something similar

*Thank you for this suggestion. This change has been made and the Author Summary (line 74) now reads:*

- Some studies suggest these children have an increased risk of congenital abnormalities, autism spectrum disorder, developmental-delay and intellectual disability.

REFERENCES
4) For in-text reference call-outs, citations should be placed within square brackets and preceding punctuation, as follows: “…asymptomatically [2,4].” 
Please check to ensure that the bibliography is listed in line with our guidance which can be found here: <https://journals.plos.org/plosmedicine/s/submission-guidelines#loc-references>

*The intext citations and bibliography have now been formatted in line with PLOS style.*

TABLE 1
5) To improve accessibility to the reader, please adjust row width/text spacing to ensure IQRs are reported in a single row or place below the relevant data point

*A re-formatted version of Table 1 has now been added to the main manuscript.*

SUPPORTING INFORMATION
6) Please ensure all figures and tables in the supporting files have abbreviations defined:
S2 TABLE 1 - Please define IVF, ITT, AEDC, NAPLAN
S4 TABLE 2b – please define “#" and  "ICSI"
S5 eMETHODS - please ensure referencing Is formatted as detailed above including in-text reference call-outs
S6 FIGURE 2 - please define NAPLAN - please check and amend throughout all supplementary files 
S10 ALL FIGURES - please define the abbreviations - LBOTE, SEIFA, NAPLAN
S16 - please define AEDC, NAPLAN, DV2
S17 TABLE 9 and 10 - please define AEDC and NAPLAN

*The requested changes to supplementary files have been made.*

SOCIAL MEDIA
6) To help us extend the reach of your research, please provide any Twitter handle(s) that would be appropriate to tag, including your own, your co-authors’, your institution, funder, or lab. Please respond to this email with any handles you wish to be included when we tweet this paper.

*The following institutional Twitter handles can be used:*

*@MercyPerinatal*

*@UniMelb*

**Comments from Reviewers:**

Reviewer #1: Many thanks to the authors for satisfactorily considering and responding to each comment in turn, suitably amending the manuscript as requested.

*We thank reviewer this reviewer for their positive comments and the time taken to review our manuscript.*

Reviewer #2: I would stand by my comments that removal of the causal claim in the title would improve the paper, I am not a fan of playing different referees of each other several of us have highlighted that the causal language may be overstated. 
"School-age outcomes among IVF-conceived children" is snappier and cuts to the chase.
*We have taken the reviewers comments on board and have adjusted the title as suggested.*

Reviewer #4: I am grateful for the opportunity to review this interesting and innovative study that seeks to estimate the total causal effect of conception by assisted reproductive technology on educational outcomes in school-age children. I was approached to provide a methodological review of the appropriateness and accuracy of the causal inference methods as well as the reporting and use of language. My review will focus primarily on these points.

Overall, I believe the study is has been well conducted and the research team should be commended for their hard work and diligence. I believe the research is scientifically sound, but I believe the quality of the manuscript could be improved with some language and presentational changes as detailed below.

*Thank you for this positive feedback.*

1)      TITLE: 'a causal inference analysis' is a vague term. I think it would be better for the study to be described more precisely as something like, 'a target trial emulation study using augmented inverse probability of treatment weighting'.

*Thank-you for this suggestion, we have removed causal language from the title. The updated title, at the recommendation of Reviewer #2, now reads: "School-age outcomes among IVF-conceived children’.*

2)      INTRODUCTION, METHODS, AND RESULTS: A causal inference approach encourages a focus on interval estimation rather than (null) hypothesis testing. I therefore find it somewhat strange that the study states a null hypothesis at the end of the introduction and goes on to report null-distribution p-values. I think it would be less jarring if the authors instead described their aim/s as something more like 'to estimate the total causal effect of conception by IVF on the risk of developmental vulnerability'. Similar language would apply in the methods and results, eg. rather than saying that 'The null hypothesis of no causal effect of IVF conception on developmental vulnerability was supported by our findings…' the study would simply report something like 'the estimated total causal effect of conception by IVF on the risk of developmental vulnerability was indistinguishable from zero (risk difference = -0.3%, 95% CI: -3.7% to 3.1%)'. 

*Thank you for this feedback. We have reframed the study aim (line 138-140) as below:*

Our study aims to estimate the total causal effect of IVF conception on school-age childhood developmental and educational outcomes using a causal inference approach and employing the necessary assumptions.

*Additionally, we have amended the results (lines 374-383 and 407-408) as follows:*

~~The null hypothesis of~~ Our findings suggest no causal effect of IVF conception on developmental vulnerability ~~was supported by our findings~~, with 13.6% of IVF-conceived children predicted to be developmentally vulnerable (<10^th^ percentile in two or more domains of the five AEDC domains) compared with 13.9% of spontaneously conceived children. The adjusted risk difference was at -0.3%, indicating that 0.3% fewer children who were conceived by IVF were developmentally vulnerable compared with those conceived spontaneously. However, the 95% confidence interval (-3.7% to 3.1%), indicate this result is indistinguishable from zero. Similarly, the adjusted relative risk showed no detectable difference in risk of developmental vulnerability, where the adjusted relative risk showed that IVF-conceived children were 3.0% less likely to be developmentally vulnerable than spontaneously conceived children (relative risk 0.97, 95% CI: 0.77 to 1.25; ~~p=0.87~~) (Table 2).

Our findings indicate the causal effect of IVF conception on overall NAPLAN z-score was indistinguishable from zero. ~~Our primary outcome was defined as~~ The predicted outcome mean z-score and was 0.013 (standard error (SE 0.024)) for IVF-conceived children and -0.016 (SE 0.002) for spontaneously conceived controls, with an adjusted mean difference of 0.030 (95% CI -0.018 to 0.077; ~~p=0.22~~) (Table 3).

3)      INTRODUCTION (lines 127-140): Although a target trial emulation study is indeed designed to 'mimic' a randomized controlled trial, I think this wording is likely to raise unfortunate associations among readers familiar with poorly-conducted propensity score analyses as 'mimicking randomised controlled trials'. I would therefore recommend toning down the language a little and simply stating that the study aimed to emulate a target trial by using the method introduced by Hernan et al with the augmented inverse probability of treatment weighting estimator. Similarly, I don't particularly like the language of achieving 'balance' in the distribution of baseline characteristic, in part because this is not what propensity score methods do (they aim to balance the outcome propensities, not the covariates). Comments about how this is 'similar to what occurs when participants are randomised' may be theoretically true, but this is unlikely to ever be achieved in practice. That said, I commend the authors for being clear about their causal aims. As outlined by Haber et al 2022 (Causal and Associational Language in Observational Health Research: A Systematic Evaluation) it is important for researchers to be clear where they seek to estimate a causal effect. It is just not necessary to 'over egg' this ambition or the methods used to achieve this aim. Thus, it is good to state an aim to 'estimate the total causal effect of conception by IVF on developmental vulnerability', and interpret the results as 'estimates of' this total causal effect, but perhaps not so good to start talking about 'mimicking randomised controlled trials'.

*Thank you for this feedback, we acknowledge the difficult balance between a clear explanation of the methodology and overstating its ability to “mimic” a target trial. We have re-worded the paragraph at the reviewers’ suggestion (lines 127-140).*

This analytical approach attempts to simulate ~~mimic~~ a randomized trial by 1) requiring an *a priori* statistical analysis protocol; 2) addressing a causal question reflecting the effect of an intervention at a specific clinical decision point on a pre-specified outcome; and 3) using inverse probability weighting via propensity score models to balance the outcome propensity differences between exposed and control populations, with the aim of producing exchangeable comparison groups, ~~similar to what occurs when participants are randomised to treatment allocation in a clinical trial~~ [27]. This allowed us to estimate the population-average effect of the mode of conception (IVF vs spontaneous conception) on childhood developmental and educational outcomes with a causal interpretation. Our study aims to estimate the total causal effect of IVF conception on school-age childhood developmental or educational outcomes using a causal inference approach and employing the necessary assumptions.

4)      METHODS: I would prefer the target trial emulation table to be presented in the main manuscript. I believe Plos Medicine is an online-only journal, so I see no reason why this should not be possible.

*We have now included the target trial emulation table (as Table 1) in the main manuscript given this request.*

5)      METHODS: There is a gap between the allocation and completion of the treatment because the conception must survive until live birth to be counted as belonging to either exposure group. In the target trial, what would happen to the pregnancies that don't result in live birth? I expect there will be an unequal chance of surviving to live birth between the in-vivo and in-vitro conceptions, creating the chance of selection bias. This ought to be acknowledged as a potential source of bias or, ideally, accounted for as competing events. 

*We agree. A paragraph on ‘live birth bias’ has now been added to the discussion section. This paragraph reads as follows (Lines 555-561):*

Furthermore, through the examination of school-based outcomes, our study was inherently designed to examine outcomes for liveborn children. “Live birth bias” as it’s known, is a recognised limitation of observational studies that investigate periconception and antenatal exposures [49]. For the purposes of this research question, the outcomes of failed conception, miscarriage, stillbirth are considered alternative endpoints and less relevant to the research question that aims to compare the school-age outcomes of children born following IVF conception with those who were conceived without assistance.

6)      METHODS: In terms of exclusion criteria, what happened to the twins and other multiple pregnancies? I cannot see them described in the exclusion criteria.

*In the Methods (line 146), our population is described as “all singleton births”. To ensure this is clear, we have added the text*:

Twins and higher order multiple births were excluded.

7)      METHODS: Regarding the target trial table, I think the authors could be more explicit in both the target trial and (in particular) the emulation study columns. I know it seems strange, but where the details of the emulation study are identical to the target trial, this really ought to be explicitly stated (either by repeating the details or simply saying 'same as for the target trial'). For example, if the inclusion criteria for the trial are 'all couples wanting to conceive and weight the ability to conceive' then the emulation column should repeat the same. In general, the emulation column does not really seem to state the expected criteria. Eg. the assignment procedures box should summarise your method for achieving conditional exchangeability and your causal contrasts box should start the target estimands. The recent paper by Matthews et al in the BMJ (Target trial emulation: applying principles of randomised trials to observational studies) offers a little help hereabout what should be mentioned in the table.

*Thank you for this important feedback. We have updated the table using these suggestions.*

8)      METHODS: I think that the supplementary methods should be included in the main manuscript, which is currently rather light on details.

*As requested, the detail surrounding handling of missing data, analysis models and model diagnostics has now been added into the main manuscript methods (from line 241-).*

9)      METHODS, RESULTS, and throughout: Since this study uses a DAG, I believe it would benefit from following the reporting recommendations outlined in Tennant et al 2021 (Use of directed acyclic graphs (DAGs) to identify confounders in applied health research: review and recommendations).

*We are familiar with this paper and our use of a DAG in this project was in keeping with the recommendations of Tennant et al. This is now explicitly stated in the manuscript (line 199-201):*

A directed acyclic graph (DAG) was created to describe the structure of the relationships between all variables and identify the adjustment variable set, in line with the methodology recommendations of Tennant et al [42].

10)     METHODS and RESULTS: The authors present 'crude' associations in the non-weighted sample. I believe these results may have been added on the instruction of a previous reviewer, as per traditional. Alas, these associations should not be reported as they are likely to be extremely biased and prone to misinterpretation. Only the best estimate of the target estimands should be reported in the main manuscript. All sub-analyses involving alternative estimators (whether unweighted logistic regression, TLME, or - if absolutely necessary - the unweighted and unconditional analyses) should be reserved for the supplementary materials.

*Crude estimates and p-values were added at the request of a previous reviewer. They have now been removed, in keeping with Reviewer #4’s recommendations and our original manuscript.*

11)     RESULTS: Null hypothesis significance testing is strongly discouraged in epidemiological analyses. The null-distribution p-values are therefore unnecessary and do not need to be reported. The authors may wish to present their E-values alongside the point estimates and confidence intervals instead.

*P-values were added at the request of previous reviewer. Point estimates and confidence intervals have been retained, as intended, as the main results. E-values have been presented as sensitivity analyses, to estimate the magnitude of the risk of unmeasured confounding.*
